# An *RPS19-*edited model for Diamond-Blackfan anemia reveals TP53-dependent impairment of hematopoietic stem cell activity

**DOI:** 10.1172/jci.insight.161810

**Published:** 2023-01-10

**Authors:** Senthil Velan Bhoopalan, Jonathan S. Yen, Thiyagaraj Mayuranathan, Kalin D. Mayberry, Yu Yao, Maria Angeles Lillo Osuna, Yoonjeong Jang, Janaka S.S. Liyanage, Lionel Blanc, Steven R. Ellis, Marcin W. Wlodarski, Mitchell J. Weiss

**Affiliations:** 1 Department of Hematology, and; 2 Department of Biostatistics, St. Jude Children’s Research Hospital, Memphis, Tennessee, USA.; 3 Institute of Molecular Medicine, Feinstein Institutes for Medical Research, Manhasset, New York, USA.; 4 Department of Biochemistry and Molecular Genetics, University of Louisville, Louisville, Kentucky, USA.

**Keywords:** Hematology, Stem cells, Gene therapy, Hematopoietic stem cells, p53

## Abstract

Diamond-Blackfan anemia (DBA) is a genetic blood disease caused by heterozygous loss-of-function mutations in ribosomal protein (RP) genes, most commonly *RPS19*. The signature feature of DBA is hypoplastic anemia occurring in infants, although some older patients develop multilineage cytopenias with bone marrow hypocellularity. The mechanism of anemia in DBA is not fully understood and even less is known about the pancytopenia that occurs later in life, in part because patient hematopoietic stem and progenitor cells (HSPCs) are difficult to obtain, and the current experimental models are suboptimal. We modeled DBA by editing healthy human donor CD34^+^ HSPCs with CRISPR/Cas9 to create *RPS19* haploinsufficiency. In vitro differentiation revealed normal myelopoiesis and impaired erythropoiesis, as observed in DBA. After transplantation into immunodeficient mice, bone marrow repopulation by *RPS19^+/−^* HSPCs was profoundly reduced, indicating hematopoietic stem cell (HSC) impairment. The erythroid and HSC defects resulting from *RPS19* haploinsufficiency were partially corrected by transduction with an *RPS19*-expressing lentiviral vector or by Cas9 disruption of *TP53*. Our results define a tractable, biologically relevant experimental model of DBA based on genome editing of primary human HSPCs and they identify an associated HSC defect that emulates the pan-hematopoietic defect of DBA.

## Introduction

Diamond-Blackfan anemia (DBA) is a rare congenital bone marrow failure disorder that typically presents in infancy as macrocytic anemia and erythroblastopenia (1, 2). DBA is associated with physical anomalies such as cleft palate, renal and cardiac defects, growth retardation, and an increased risk for certain cancers (3, 4). Although hypoplastic anemia is the dominant feature in children, bone marrow hypocellularity, pancytopenia, and immunodeficiency can develop in older patients, suggesting hematopoietic stem cell (HSC) impairment (5, 6). Classical DBA is caused by germline heterozygous loss-of-function mutations in 1 of 20 small- or large-subunit ribosomal protein (RP) genes, leading to defective ribosome biogenesis and/or function. Less frequently, mutations in *GATA1* (7), *EPO* (8), *ADA2* (9), and *TSR2* (10) cause DBA-like hypoplastic anemia. The most common DBA gene is *RPS19*, with mutations detected in approximately 25% of patients. The next most commonly mutated genes are *RPL5* (~7%), *RPS26* (~7%), and *RPL11* (~5%) (1). Current therapies for DBA include chronic red blood cell transfusions with iron chelation; glucocorticoids, which drive the expansion of erythroid progenitors; and allogeneic hematopoietic stem cell transplantation (HSCT), all of which are associated with major toxicities.

The mechanisms of DBA-associated erythroid failure are not fully understood. Analysis of patient hematopoietic stem and progenitor cells (HSPCs) has revealed defects in erythroid progenitor expansion with pathological apoptosis of erythroid progenitors (1, 11–14). Potential explanations include impaired global translation (15, 16); selectively impaired translation of transcripts essential for erythropoiesis such as *BAG1* (17), *CSDE1* (17), and *GATA1* (18, 19); build-up of cytotoxic free heme due to reduced translation of globin proteins (20, 21); and activation of TP53 (18, 22, 23). Studies in mouse models and cancer cell lines suggest that a deficiency of some RPs can destabilize ribosome assembly, leading to the accumulation of free RPs, including RPL5 and RPL11, which sequester MDM2 and inhibit its ubiquitin ligase activity that promotes TP53 degradation (24, 25). Limited studies have suggested additional mechanisms of DBA-associated erythroid failure, including hyperactivation of Nemo-like kinase (26) and altered autophagy (27).

While the mechanisms of erythroid failure in DBA are not fully defined, even less is known about the impact of RP haploinsufficiency on HSPCs. Patients with DBA are predisposed to developing age-related pancytopenia (5, 6), myelodysplastic syndrome, and myeloid leukemia (3, 4, 28). Although *Rps19^+/–^* mice exhibit no hematopoietic abnormalities (29), further reduction of *Rps19* by an in vivo–expressed short hairpin RNA causes *Trp53*-dependent anemia and HSC exhaustion (29). DBA patient HSPCs were shown to have an intrinsic qualitative defect, as revealed by reduced clonogenic output in long-term culture-initiating assays (5). Two studies have examined HSPCs from patients with DBA after xenotransplantation into immunodeficient mice. One of these studies showed that engraftment of *RPS19*-mutated CD34^+^ HSPCs from patients with DBA was improved by transduction with an *RPS19*-expressing lentiviral vector (LV), but healthy donor HSPCs were not examined as controls (30). In another report, HSPCs from patients with DBA engrafted normally in NOD/SCID mice but erythropoiesis was reduced (31). However, the engraftment levels of DBA and control HSPCs were less than 1%.

Investigations of potential HSPC defects in DBA are constrained by the limited availability of patient samples for research and the lack of a human cellular model that faithfully recapitulates the disease. Zebrafish and mouse models do not precisely recapitulate human DBA (18, 32). Transducing normal donor CD34^+^ cells with LVs expressing short hairpin RNAs to deplete RP genes causes impaired erythropoiesis in vitro and has been a useful tool in DBA research (10, 33, 34). However, it is difficult to achieve precise haploinsufficiency to recapitulate a disease that is ostensibly dependent on the dosage of the affected genes (27). We and others have previously demonstrated an erythroid defect in induced pluripotent stem cells derived from patients with DBA (27, 35), but these cells are currently of limited utility for studying HSC properties. Therefore, we sought to model DBA and study the effects of RP haploinsufficiency on adult-type definitive HSCs. We used CRISPR/Cas9 to disrupt *RPS19* in healthy donor CD34^+^ HSPCs. In vitro differentiation of this population revealed impaired erythropoiesis but normal myelopoiesis, recapitulating the canonical findings of DBA (1). By using a comprehensive flow cytometry panel (36), we showed that this defect is associated with increased apoptosis of colony-forming unit–erythroid (CFU-E) progenitors. Additionally, xenotransplantation studies revealed defective bone marrow repopulation by *RPS19^+/–^* HSCs. Both defects were rescued by transduction with an *RPS19*-encoding LV or genetic suppression of *TP53*. Our studies define a tractable experimental model for *RPS19*-mutated DBA using primary human HSPCs and show that *RPS19* haploinsufficiency impairs HSCs through a TP53-dependent mechanism.

## Results

### CRISPR/Cas9 disruption RPS19 in CD34^+^ HSPCs.

**Figure 1:**
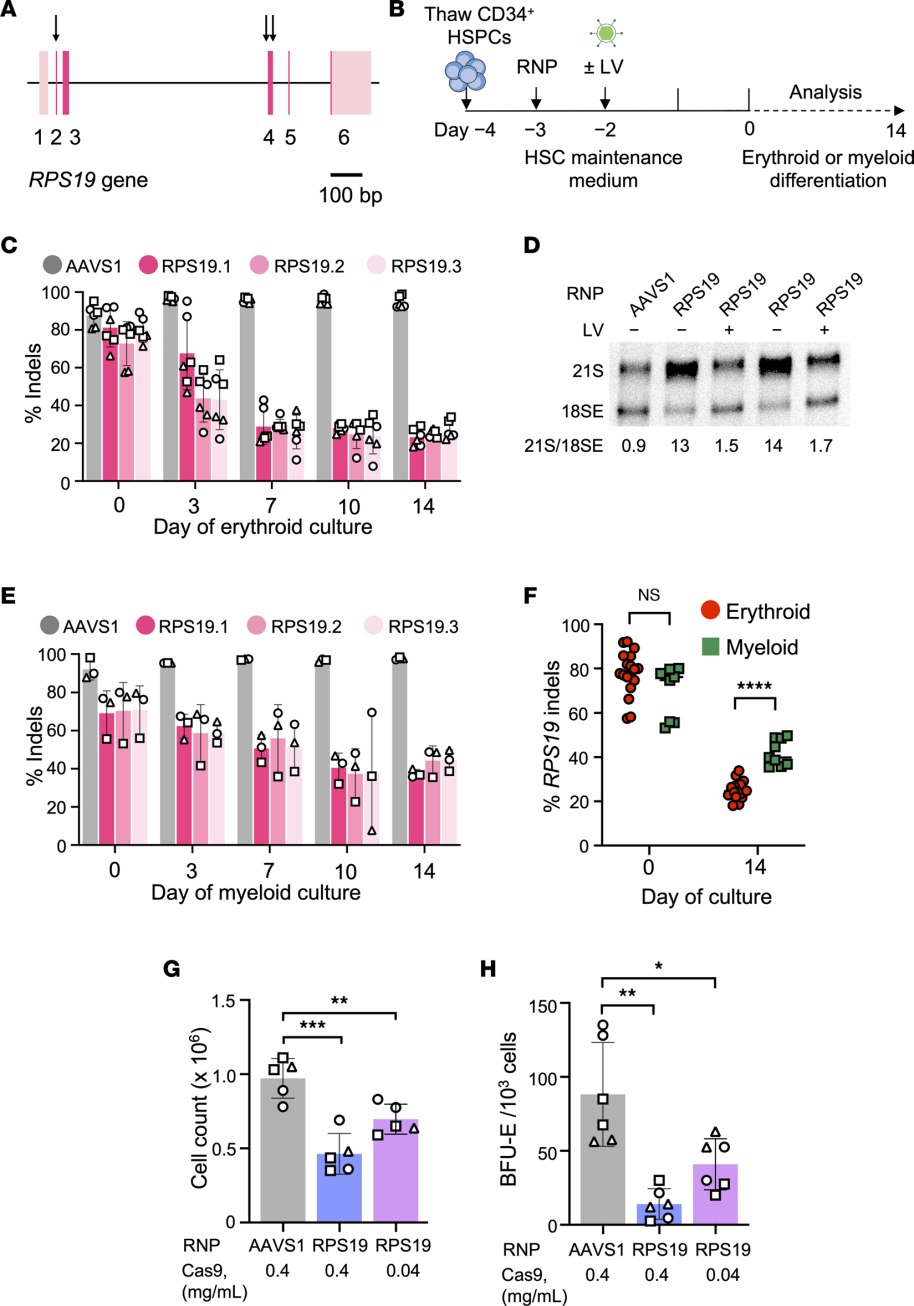
Cas9 disruption of the *RPS19* gene in CD34^+^ HSPCs. (**A**) Diagram of *RPS19*, including exons 1–6, with coding regions in a darker shade. Arrows show regions targeted by 3 single-guide RNAs (sgRNAs). (**B**) Experimental protocol for the in vitro studies in Figures 1–3. On day −3, healthy donor CD34^+^ HSPCs were edited by electroporation with ribonucleoprotein (RNP) complex consisting of Cas9-3×NLS plus sgRNAs targeting *AAVS1* (as a control) or *RPS19*. In some experiments, cells were transduced with an *RPS19*-expressing or control lentiviral vector (LV) on day −2. The frequency of on-target insertion-deletion (indel) mutations was determined by next-generation sequencing (NGS) on day 0, and cells were switched to medium containing cytokines for erythroid (EPO, SCF, IL-3) or myeloid (SCF, TPO, G-CSF, GM-CSF) differentiation. (**C**) Indel frequency versus time in erythroid medium. (**D**) Northern blot analysis of RNA from gene-edited HSPCs using ITS1 probe. (**E**) Indel frequency versus time in myeloid medium. (**F**) *RPS19* indel frequency on days 0 and 14 of erythroid and myeloid culture, from **C** and **E**. Data represent a total of 18 experiments for erythroid differentiation and 9 experiments for myeloid differentiation, using 3 different sgRNAs and 3 different CD34^+^ cell donors. (**G**) CD34^+^ HSPCs (1 × 10^6^) were edited with 0.4 or 0.04 mg/mL RNP (Cas9 component) containing *AAVS1* or *RPS19.1* sgRNA. Three days later (day 0), live cells were quantified with a NucleoCounter NC-200 automated cytometer (ChemoMetec). (**H**) Burst-forming unit–erythroid (BFU-E) colonies per 1000 CD34^+^ HSPCs. All bar charts show the data as the mean ± SD, with each symbol representing data from different CD34^+^ cell donors. *P < 0.05; **P < 0.01; ***P < 0.001; ****P < 0.0001 (unpaired, 2-tailed Student’s *t* test). *P* values were adjusted for multiple comparison in **F**, **G**, and **H** by the Holm-Bonferroni method.

We designed 3 different sgRNAs targeting *RPS19* exon 2 or 4, and 1 sgRNA targeting the “safe harbor locus” *AAVS1* (Figure 1A and Supplemental Table 1; supplemental material available online with this article; https://doi.org/10.1172/jci.insight.161810DS1). Healthy donor peripheral blood mobilized CD34^+^ HSPCs were electroporated with a ribonucleoprotein (RNP) complex consisting of Cas9 and sgRNA, and then incubated in HSPC maintenance medium (Figure 1B). For some studies, cells were transduced with a third-generation, self-inactivating LV in which the EF1α core promoter drives the expression of a bicistronic mRNA encoding *RPS19* and *GFP* separated by the ribosome-skipping P2A sequence (Supplemental Figure 1) (37, 38). Three days after electroporation (day 0), the on-target indel frequencies were determined by next-generation sequencing (NGS) and the cells were transferred to erythroid or myeloid differentiation medium (Figure 1B). All targeting RNPs resulted in high frequencies of indels (Figure 1C), most of which caused frameshift mutations predicting loss of protein function (Supplemental Figure 2, A and B, and Supplemental Table 2). Western blot analysis of cells targeted with sgRNA RPS19.1 showed RPS19 protein to be reduced by approximately 40% (Supplemental Figure 3, A and B). Transduction of *RPS19-*targeted HSPCs with *RPS19-GFP* LV followed by Western blot analysis showed efficient cleavage at the P2A site and expression of LV-encoded RPS19, which is fused to the 20–amino acid P2A peptide (Supplemental Figure 3C). Deficiency of RPS19 impairs processing of the *18S* rRNA precursor, resulting in an increased ratio of *21S*/*18SE* rRNA intermediates (39). This defect was apparent in *RPS19*-targeted CD34^+^ cells, compared with those targeted at the control *AAVS1* locus (Figure 1D, lanes 1, 2, and 4), and rescued by transduction with *RPS19-GFP* LV (Figure 1D, lanes 3 and 5).

During in vitro erythroid differentiation of RNP-treated CD34^+^ cells, *AAVS1* indel frequencies were stable over time, whereas *RPS19* indels declined, suggesting dropout of *RPS19-*deficient cells (Figure 1C). The *RPS19* indel frequency also decreased over time in myeloid culture, although to a lesser extent than in erythroid culture (Figure 1, E and F), suggesting that erythroid cells are more sensitive to *RPS19* loss than are myeloid cells in vitro. All 3 *RPS19* sgRNAs produced similar results, indicating that impaired erythropoiesis is due to *RPS19* disruption and not off-target effects. The sgRNA RPS19.1 was used in subsequent experiments because it is predicted to have fewer off-target DNA cleavage sites (40). Compared with *AAVS1* targeting, *RPS19* disruption resulted in a greater than 50% reduction in live cells at 3 days after electroporation (*P* < 0.001) (Figure 1G) and generated more than 80% fewer burst-forming unit–erythroid (BFU-E) colonies (*P* *<* 0.01) (Figure 1H). This could be partly due to some cells having biallelic *RPS19* disruption, which are likely nonviable (41). Reducing the dose of RNP from 0.4 to 0.04 mg/mL Cas9 component resulted in improved survival of HSPCs, most likely due to fewer biallelic edits (Figure 1G), an increased number of BFU-E colonies (Figure 1H) and lower indel frequencies (Supplemental Figure 4A). Hence, the lower dose of RPS19.1 RNP (0.04 mg/mL) was used in subsequent experiments to minimize cell loss caused by biallelic edits.

### RPS19-targeted HSPCs exhibit an erythroid progenitor maturation defect.

**Figure 2:**
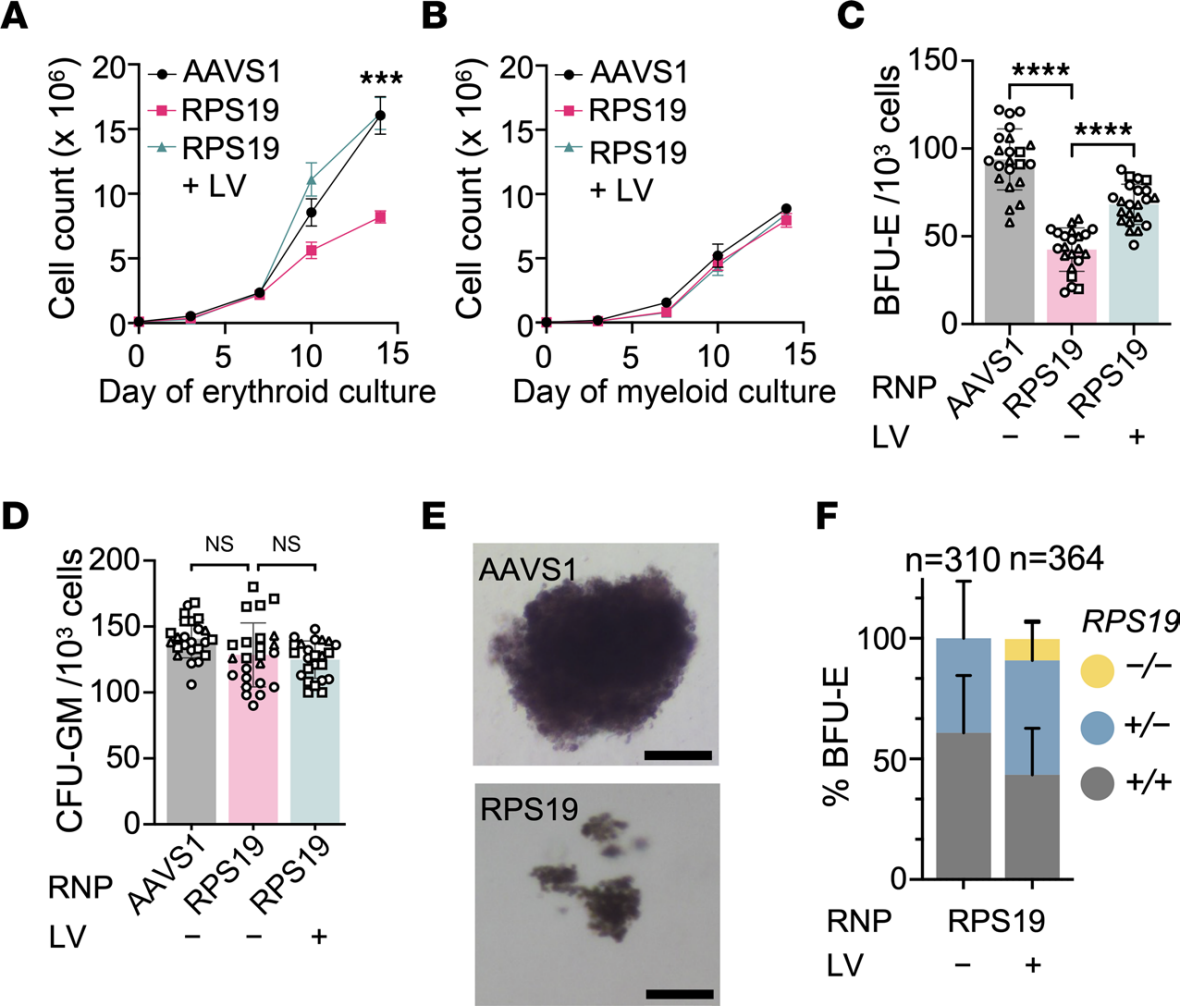
*RPS19^+/–^* CD34^+^ HSPCs exhibit a selective erythroid defect. A total of 1 × 10^6^ healthy donor CD34^+^ HSPCs were electroporated with 0.04 mg/mL Cas9-3×NLS plus *AAVS1* (as control) or *RPS19.1* sgRNA on day −3, transduced with *RPS19* LV on day −2, and switched to erythroid or myeloid medium on day 0. (**A**) Total live cell number versus time in erythroid culture. Each data point represents the mean ± SD of 3 biological replicate studies with different CD34^+^ cell donors. Asterisks indicate significant differences between RPS19 RNP–treated cells and either AAVS1 RNP–treated cells or RPS19 RNP–treated cells rescued with *RPS19* LV. (**B**) Live cell number versus time in myeloid culture, shown as described for **A**. (**C**) A total of 500–1000 CD34^+^ cells were seeded into 1 mL of methylcellulose medium with erythroid cytokines. Burst-forming unit–erythroid (BFU-E) colonies were enumerated 14 days later. (**D**) Granulocyte-macrophage colony–forming units (CFU-GMs) per 1000 CD34^+^ cells seeded as described in **C**. (**E**) Images of representative BFU‑E colonies generated after treatment with AAVS1 or RPS19 RNPs. The images were captured using a Nikon DS QI2 camera on a Nikon Eclipse NI microscope. Scale bars: 50 μm. (**F**) Genotype distributions for BFU-E colonies generated by *RPS19*-edited CD34^+^ HSPCs with and without gene rescue with *RPS19* LV. *n* = total colonies analyzed from biological replicate experiments using 4 different CD34^+^ HSPC donors. Bar charts show the mean ± SD of each genotype, with each symbol representing data from different CD34^+^ cell donors. ***P < 0.001; ****P < 0.0001 by linear mixed-effects model (**A**) or unpaired, 2-tailed Student’s *t* test (**C** and **D**). *P* values were adjusted for multiple comparison in **C** and **D** by the Holm-Bonferroni method. NS, not significant.

We compared the phenotypes of *RPS19- and AAVS1-*targeted HSPCs, beginning at the initiation of erythroid or myeloid differentiation (Figure 1B; day 0). Treatment with RPS19 RNP caused an approximately 50% reduction in cell number after 14 days in erythroid culture (*P* < 0.001) but no reduction in cell number in myeloid culture (Figure 2, A and B). Transducing *RPS19*-targeted HSPCs with *RPS19-GFP* LV rescued the defect in erythroid cell expansion but had no effect on the growth of the same cells in myeloid medium. Dropout of *RPS19* indels in erythroid culture was reduced by transduction with *RPS19-GFP* LV (Supplemental Figure 4B), suggesting that LV gene rescue improves the survival of *RPS19*-disrupted cells. In methylcellulose medium, *RPS19-*targeted HSPCs generated 55% fewer BFU-E colonies compared with control *AAVS1-*targeted cells (*P* < 0.0001) but there was no change in the number of granulocyte-macrophage colony–forming units (CFU-GMs) (Figure 2, C and D). The reduction in BFU-E colonies generated by *RPS19*-disrupted HSPCs was partially rescued by transduction with *RPS19-GFP* LV (*P* < 0.0001) (Figure 2C). Many BFU-E colonies generated from *RPS19-*targeted HSPCs were smaller and contained fewer cells on average than control colonies (*P* < 0.05) (Figure 2E and Supplemental Figure 4C). Therefore, *RPS19-*targeted HSPCs exhibit selectively impaired erythropoiesis in vitro with intact myelopoiesis, as observed in bone marrow cells from patients with DBA (42, 43). We analyzed on-target indels in clonal BFU‑E colonies derived from *RPS19*-targeted HSPCs. Editing with low-dose RNP (0.04 mg/mL) generated roughly equal numbers of *RPS19^+/−^* and *RPS19^+/+^* colonies (Figure 2F). No *RPS19^−/−^* colonies were detected, confirming their nonviability. However, rescue with *RPS19-GFP* LV supported the survival of *RPS19^−/−^* erythroid colonies (Figure 2F).

**Figure 3:**
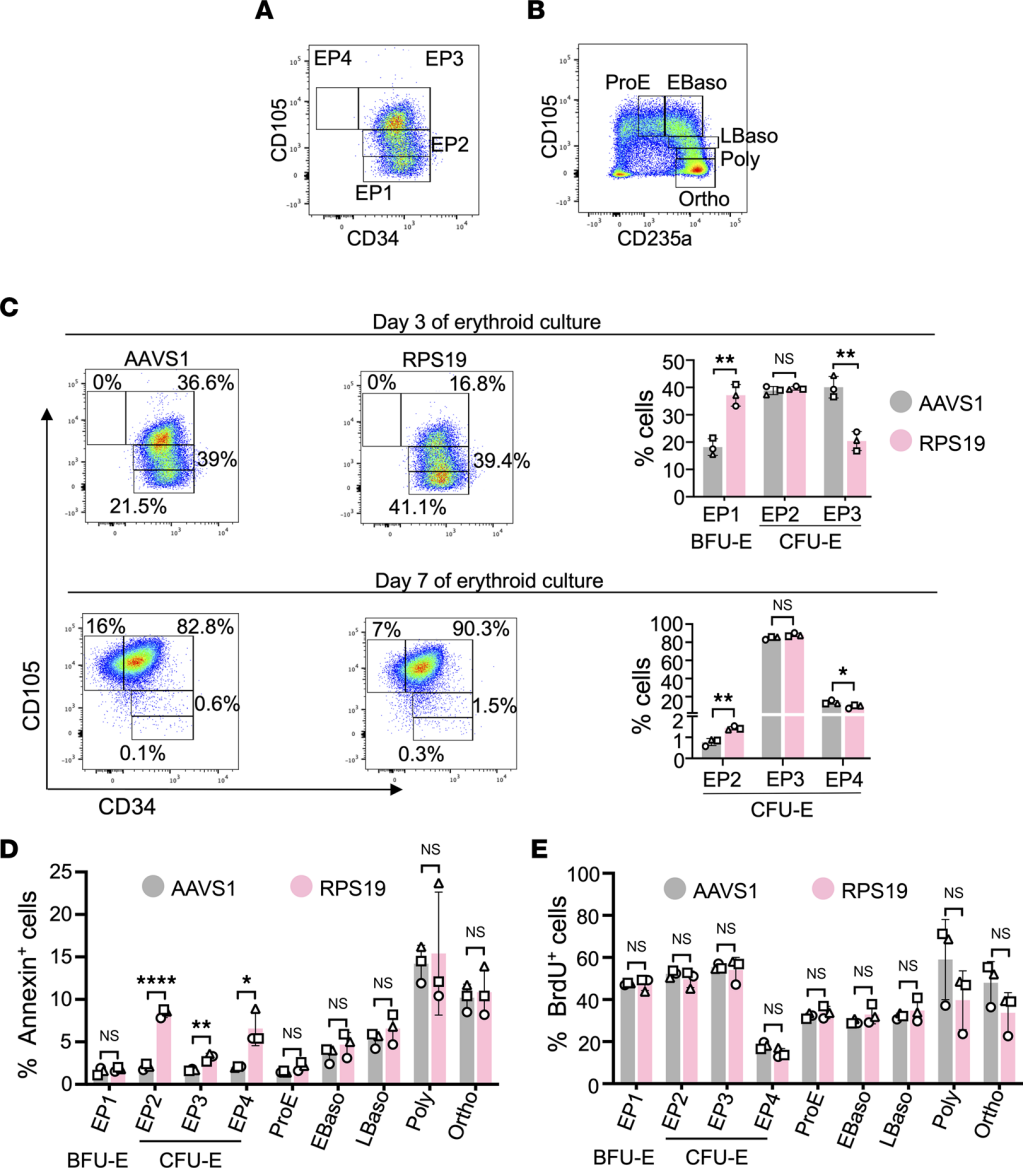
*RPS19* haploinsufficiency impairs erythroid progenitor maturation. (**A**) Gating strategy for erythroid progenitor (EP) stages 1–4 (36). (**B**) Gating strategy for terminal erythroid differentiation (36). (**C**) AAVS1 RNP–treated or RPS19 RNP–treated cells from erythroid medium were analyzed by flow cytometry on days 3 and 7, using the schematic shown in panels **A** and **B** and in Supplemental Figure 5A. Representative flow cytometry plots for EP stages based on expression of CD34 and CD105 are shown. (**D**) Percentage of annexin V^+^ cells during erythroid differentiation. (**E**) Percentage of BrdU^+^ cells after a 45-minute pulse. All bar charts show the data as the mean ± SD, with each symbol representing data from different CD34^+^ cell donors. *P < 0.05; **P < 0.01; ****P < 0.0001 (unpaired, 2-tailed Student’s *t* test). ProE, proerythroblasts; EBaso, early basophilic erythroblasts; LBaso, late basophilic erythroblasts; Poly, polychromatic erythroblasts; Ortho, orthochromatic erythroblasts.

To pinpoint the developmental stage of erythropoiesis that was impaired by *RPS19* haploinsufficiency, we analyzed edited HSPCs undergoing erythroid differentiation by immuno-flow cytometry with an antibody panel that distinguishes 7 distinct erythroid progenitors and precursors with successively increased maturation and reduced proliferative capacity: BFU-E (EP 1), CFU-E (EP2-4), proerythroblast (ProE), early basophilic erythroblast (Ebaso), late basophilic erythroblast (Lbaso), polychromatophilic erythroblast (Poly), and orthochromatic erythroblast (Ortho) (Figure 3, A and B, and Supplemental Figure 5A) (36). At day 3 of differentiation, *RPS19-*targeted erythroid populations exhibited a partial developmental block at the transition from BFU-E to CFU-E (Figure 3C), similar to the erythropoietic block observed in patients with DBA (12, 14, 23). Moreover, *RPS19-*targeted CFU‑E populations contained an increased proportion of apoptotic (annexin V^+^) cells when compared with control CFU-E cells, with no change in proliferation measured by BrdU uptake (Figure 3, D and E). At differentiation day 14, there were small but significant differences in the proportions of polychromatophilic and orthochromatic erythroblasts, with no differences in apoptosis, proliferation, or morphology (Figure 3, D and E, and Supplemental Figure 5, B and C), most likely representing mildly impaired maturation of late-stage *RPS19^+/–^* erythroid precursors caused by an unknown mechanism. In contrast to the impaired erythropoiesis observed in *RPS19*-targeted HSPCs, there were no detectable differences in myeloid maturation (Supplemental Figure 6, A and B). Together, our data show that Cas9-mediated heterozygous disruption of *RPS19* in primary human HSPCs reproduces numerous pathologic features of DBA erythropoiesis.

### Persistent TP53 activity in RPS19^+/−^ HSPCs.

**Figure 4:**
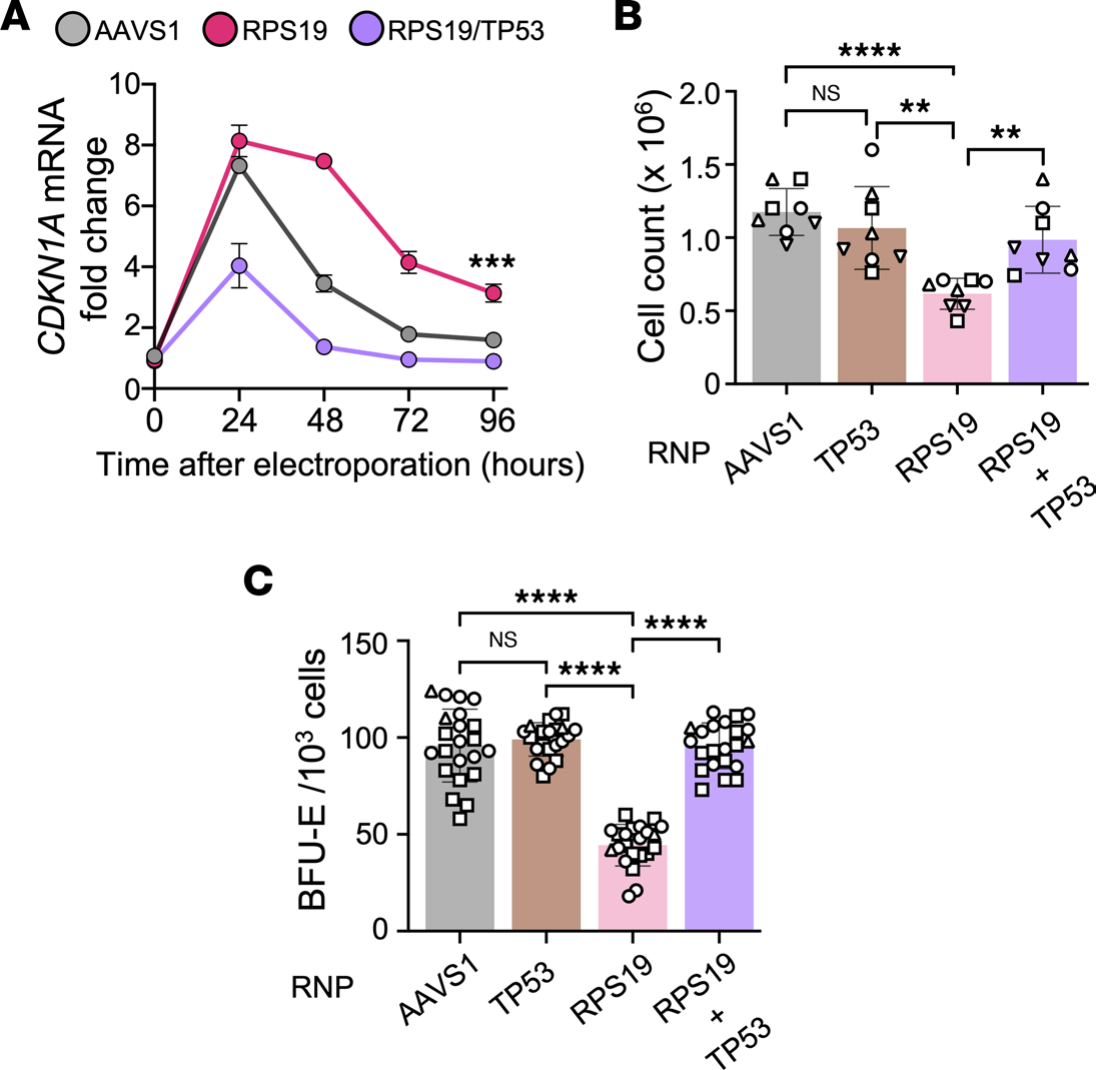
TP53 impairs erythroid development of *RPS19^+/–^* HPSCs. A total of 1 × 10^6^ healthy donor CD34^+^ HSPCs were edited with RNPs targeting *RPS19* and/or *TP53* according to the protocol in Figure 1B. (**A**) Expression level of the TP53 target gene *CDKN1A* versus time after electroporation, relative to the level in unedited cells. *CDKN1* mRNA levels were quantified by RT-ddPCR and normalized to *GAPDH*. Each data point represents the mean ± SD of 3 biological replicate experiments using CD34^+^ cells from different donors. Asterisks indicate significant difference between cells treated with RPS19 RNP and AAVS1 RNP. (**B**) Viable cell counts at 3 days after electroporation. (**C**) BFU-E colonies per 10^3^ CD34^+^ HSPCs. Bar charts in **B** and **C** show the mean ± SD; each symbol represents a different CD34^+^ cell donor. **P < 0.01; ***P < 0.001; ****P < 0.0001 by linear mixed-effects model (**A**) or unpaired, 2-tailed Student’s *t* test (**B** and **C**). *P* values were adjusted for multiple comparison in **B** and **C** by the Holm-Bonferroni method.

Increased TP53 activity can arise from ribosomal stress and probably contributes to anemia in DBA, although specific details are unresolved (18, 22). For example, the pattern of TP53 activation in HSPCs may vary with different RP mutations (44–46) and in different species (29). We treated HSPCs with RPS19 or AAVS1 RNP and then measured expression of the TP53 transcriptional target *CDKN1A* by reverse-transcribed digital droplet PCR (RT-ddPCR) (Figure 4A). Treatment with AAVS1 RNP caused transient induction of *CDKN1A* mRNA, consistent with a TP53 DNA damage response arising from Cas9-induced double-stranded DNA breaks (47). In contrast, *RPS19* editing caused stronger and more sustained increases in the expression of *CDKN1A* mRNA and protein, which were eliminated by codisruption of *TP53* (48) (Figure 4A and Supplemental Figure 7, A and B). Additionally, treating HSPCs with RPS19 RNP caused impaired cell recovery after 3 days and reduced BFU-E colony formation, both of which were rescued by homozygous disruption of *TP53* (Figure 4, B and C, and Supplemental Figure 7C). However, loss of *TP53* did not support the formation of *RPS19^−/−^* BFU-E colonies (Supplemental Figure 7D). These findings show that *RPS19* haploinsufficiency causes TP53 activation in HSPCs, leading to impaired erythroid development.

### Defective bone marrow repopulation by RPS19^+/−^ HSPCs.

**Figure 5:**
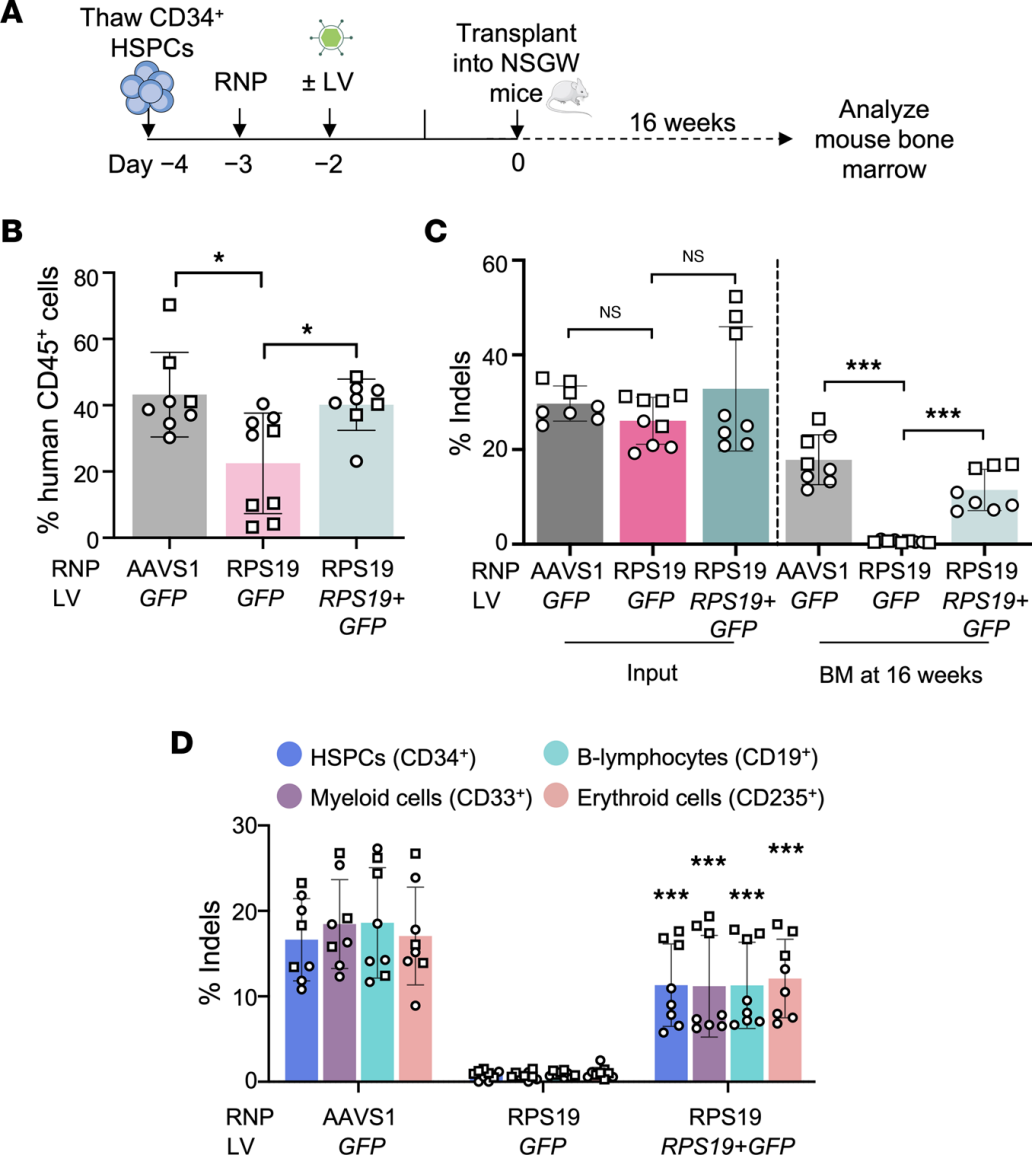
*RPS19^+/–^* HSPCs exhibit defective bone marrow repopulation after xenotransplantation. (**A**) Experimental scheme for xenotransplantation studies. CD34^+^ HSPCs (*n* = 2 different donors) were edited with the indicated RNPs followed by transduction with LVs encoding *GFP* or *RPS19* plus *GFP* at a multiplicity of infection (MOI) of 20. A total of 4 × 10^5^ to 5 × 10^5^ live cells were transplanted into NSGW mice (*n* = 8–9 mice), which were euthanized and analyzed after 16–18 weeks. (**B**) Percentage of human CD45^+^ cells in recipient bone marrow. Data were analyzed by 1-way ANOVA test and pairwise testing was performed with Tukey’s adjustment for multiple comparisons. (**C**) Indel frequency in input donor CD34^+^ HSPCs on day 0 and in donor-derived cells in recipient mouse bone marrow at 16–18 weeks after xenotransplantation. Data were analyzed by Wilcoxon’s rank-sum test. (**D**) Indel frequencies in CD34^+^ HSPC donor-derived hematopoietic lineages purified from recipient mouse bone marrow by flow cytometry using the indicated antibodies. The frequency of human T cells in recipient bone marrow was <0.01% (not shown). Data were analyzed by unpaired, 2-tailed Student’s *t* test. Asterisks indicate significant differences between *RPS19*-edited HSPCs transduced with *RPS19* plus *GFP* LV versus *GFP* LV. All charts show the mean ± SD, with each dot representing an individual mouse and each symbol representing a different CD34^+^ cell donor. *P* values were adjusted for multiple comparison by the Holm-Bonferroni method unless specified otherwise. *P < 0.05; ***P < 0.001.

To determine whether *RPS19* haploinsufficiency impaired HSPCs, we transplanted CD34^+^ HSPCs treated with RNPs targeting *AAVS1* or *RPS19* into immunodeficient NSGW mice (Figure 5A). For some studies, gene-edited HSPCs were transduced with *RPS19-GFP* LV or control *GFP* LV before xenotransplantation. Recipient mice were euthanized for analysis by flow cytometry and indel quantification after 16 weeks (Supplemental Figure 8A). Transplantation with *RPS19-*targeted HSPCs resulted in lower levels of bone marrow chimerism than control *AAVS1*-targeted HSPCs, as measured by the expression of human CD45 (Figure 5B). This abnormality was corrected by transduction with *RPS19-GFP* LV. In donor cells treated with AAVS1 RNP plus control *GFP* LV, the indel frequency dropped from 29.7% ± 3.7% (SD) at 3 days after editing (input) to 17.8% ± 5.3% at 16 weeks after transplantation (a 40% reduction) (Figure 5C). In contrast, in cells treated with RPS19 RNP plus *GFP* LV, the indel frequency dropped from 26.1% ± 5.0% in input cells to 0.6% ± 0.3% after 16 weeks (a 98% reduction), which is below the limit of detection. Thus, in this competitive repopulation assay, unedited *RPS19^+/+^* HSCs engraft the bone marrow, whereas engraftment by *RPS19^+/−^* edited HSCs was markedly impaired. This defect was partially alleviated by transducing targeted HSPCs with *RPS19-GFP* LV (*P* < 0.001) (Figure 5C). The input cells had an *RPS19-GFP* vector copy number (VCN) of 2/diploid genome (dg) (Supplemental Figure 8B) and a transduction efficiency of approximately 60%, as measured by the percentage of GFP^+^ cells (Supplemental Figure 8C). At 16 weeks, the overall VCN was 1/dg with approximately 40% GFP^+^ cells (Supplemental Figure 8, B and C), suggesting that a VCN of approximately 2/dg was sufficient to rescue the bone marrow repopulation defect of *RPS19*^+/−^ HSPCs. In flow cytometry–purified human donor HSC–derived myeloid (CD33^+^), erythroid (CD235a^+^), B lymphocyte (CD19^+^), and HSPC (CD34^+^) lineages, dropout of *RPS19* indels was noted to occur in a similar fashion to that of bulk mouse bone marrow cells (Figure 5D), although the percentage of human cells in each lineage across the 3 groups was similar (Supplemental Figure 8D). Therefore, *RPS19* haploinsufficiency confers a bone marrow repopulation defect to HSCs. Rescue of bone marrow engraftment by *RPS19-GFP* LV indicates that the defect is due to RPS19 deficiency, rather than nonspecific toxicities of genome editing.

### TP53 disruption restores engraftment of RPS19^+/−^ HSPCs.

**Figure 6:**
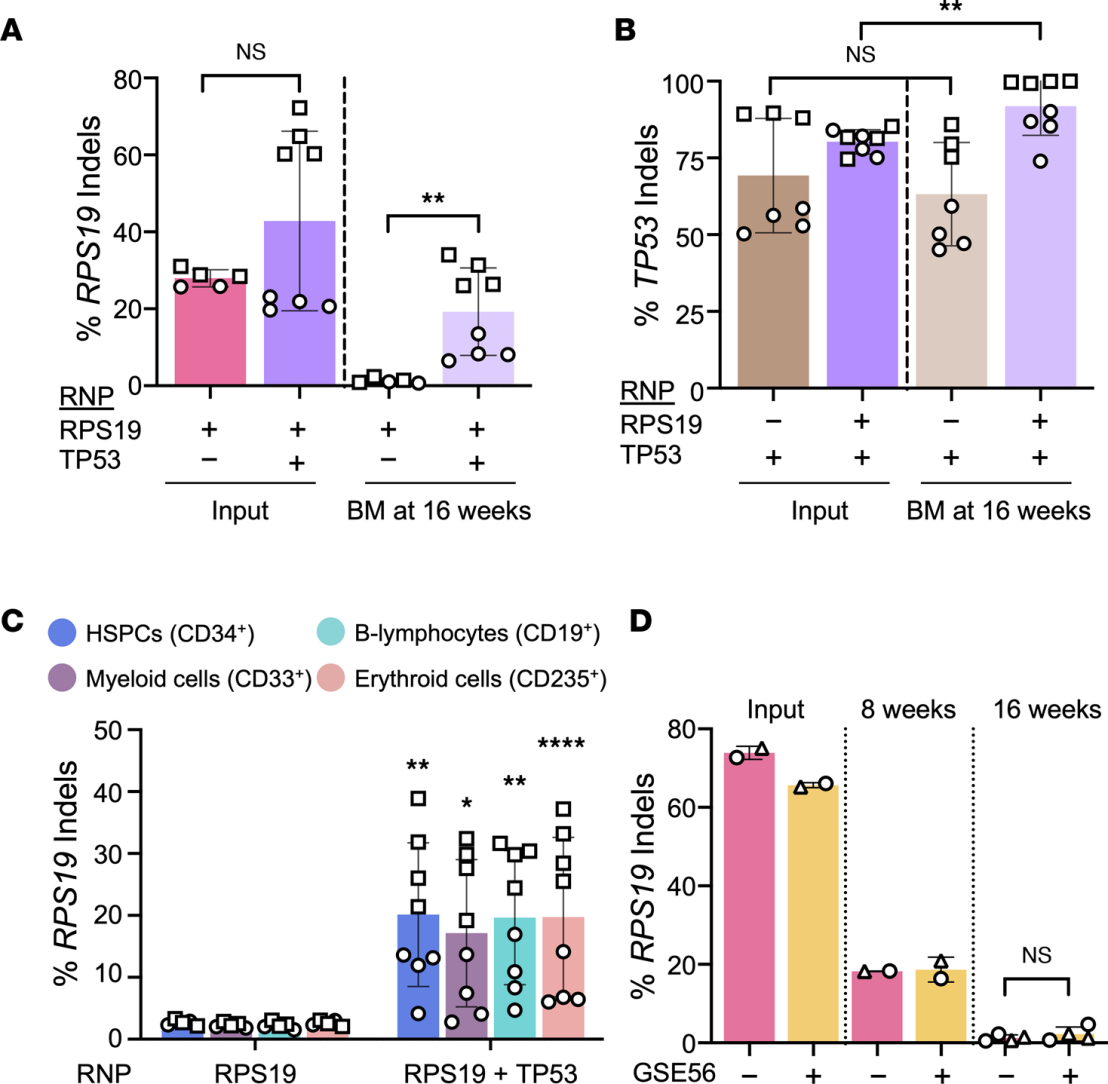
TP53 activation impairs engraftment of *RPS19*^+/–^ HSPCs. CD34^+^ HSPCs (*n* = 2 different donors) were edited with RPS19 and/or TP53 RNP and analyzed by xenotransplantation (*n* = 5–8 mice) according to the protocol shown in Figure 4A. (**A**) *RPS19* indel frequency in input CD34^+^ HSPCs on day 0 and in bulk bone marrow 16 weeks after xenotransplantation. Data were analyzed by Wilcoxon’s rank-sum test. (**B**) *TP53* indel frequencies. (**C**) *RPS19* indel frequency in human donor CD34^+^ HSPC–derived hematopoietic lineages purified from recipient mouse bone marrow by flow cytometry. All charts show the data as the mean ± SD, with each dot representing an individual mouse and each symbol a different CD34^+^ cell donor. Asterisks indicate significant differences between *RPS19*-disrupted versus *RPS19*- and *TP53*-disrupted cells. (**D**) *RPS19* indel frequency in input CD34^+^ HSPCs, treated with RPS19 RNP with and without GSE56 mRNA, 3 days after electroporation and in bulk bone marrow at 8 weeks and 16 weeks after transplantation. Data in **B**–**D** were analyzed by unpaired, 2-tailed Student’s *t* test. *P* values were adjusted for multiple comparison by the Holm-Bonferroni method. *P < 0.05; **P < 0.01; ****P < 0.0001.

To investigate whether the repopulation defect in *RPS19^+/−^* HSCs is TP53 dependent, CD34^+^ HSPCs were edited with RNP targeting one or both genes and then analyzed by xenotransplantation. After editing *RPS19* alone, the indel frequency dropped by 95%, from 27.9% ± 2.2% in input cells to below the limit of detection (1.2% ± 0.7%), as expected (Figure 6A). After *RPS19*/*TP53* multiplex editing, the *RPS19* indel frequency declined by only 55%, which represented an approximately 16-fold increase in *RPS19* indels, as compared with those in cells treated with RPS19 RNP alone (*P* < 0.01) (Figure 6A and Supplemental Figure 9A). The *TP53* indel frequencies were similar in input cells and at 16 weeks after transplantation, suggesting that *TP53* disruption alone conferred no major selective advantage to HSPCs (Figure 6B). However, the *TP53* indel frequency increased slightly in *RPS19*/*TP53*-edited donor cells, likely reflecting a survival advantage of *TP53-*edited *RPS19^+/−^* cells over *TP53^+/+^ RPS19^+/−^* HSPCs (Figure 6B). Similarly, *TP53* disruption prevented dropout of *RPS19* indels in flow cytometry–purified, human donor HSC–derived myeloid, erythroid, B lymphocyte, and HSPC lineages (Figure 6C), although the percentage of human cell lineages was unchanged between the groups (Supplemental Figure 9B).

To determine whether transient suppression of TP53 during the genome editing process can rescue the bone marrow engraftment defect of *RPS19^+/−^* HSCs, we cotransfected CD34^+^ cells with RPS19 RNP and GSE56 mRNA, which encodes a dominant negative form of TP53 that can enhance the engraftment of gene-edited human HSPCs (49, 50). GSE56 mRNA inhibited TP53 activation after *RPS19* editing, as evidenced by a significant reduction in *CDKN1A* mRNA after electroporation (*P* < 0.001; Supplemental Figure 9C). However, transient suppression of TP53 activity did not rescue the bone marrow engraftment defect of *RPS19^+/–^* HSPCs at 16 weeks after xenotransplantation (Figure 6D). Moreover, while *RPS19* indels were not detected at this time point, relatively low levels of *RPS19^+/–^* HSPCs were detected in recipient bone marrow at 8 weeks after transplantation (Figure 6D). Thus, Cas9-induced *RPS19* haploinsufficiency does not eliminate early engraftment following xenotransplantation. Rather, *RPS19^+/−^* HSCs are outcompeted over time by *RPS19^+/+^* HSCs in a TP53-dependent manner.

## Discussion

Modern genetics has identified heterozygous loss-of-function RP gene mutations as the major cause of DBA (1, 9), yet we do not fully understand how these mutations impair hematopoiesis, in part because patient samples are difficult to obtain. Our study has addressed this problem by creating and validating a primary HSPC–based model of human DBA. We showed that Cas9-induced *RPS19* haploinsufficiency in normal donor CD34^+^ HSPCs resulted in increased apoptosis of early CFU-E progenitors. These findings are consistent with studies of DBA patient cells (1, 11, 14, 22) and validate our experimental model. Potential mechanisms for selective apoptosis of CFU-E in DBA include impaired translation of transcription factor GATA1, accumulation of toxic free heme, and premature reduction in ribosome biogenesis leading to TP53 activation (1, 19, 20, 22, 51). Additionally, our findings provide insights into prior clinical and laboratory observations suggesting that HSC maintenance is impaired in DBA (2, 52–54). Specifically, the current study provides the first definitive evidence to our knowledge that *RPS19* haploinsufficiency causes a TP53-dependent defect in bone marrow repopulation of human HSCs. We observed the same erythropoietic and HSC defects after disrupting *RPS19* in CD34^+^ HSPCs from multiple healthy donors, and the abnormalities were rescued by lentiviral transfer of *RPS19* cDNA.

While the erythroid defect in *RPS19-*mutated DBA is thought to be TP53 mediated (13, 22), activation of TP53 has not been previously described in *RPS19^+/–^* human HSPCs. Our findings suggest that the panhematopoietic defect seen in some older DBA patients (5, 6) is caused by aberrant TP53 activation in HSPCs. TP53 activation can alter cell division, survival/apoptosis, metabolism, DNA repair, autophagy, and protein translation to inhibit stem cell pluripotency and promote differentiation (55, 56). In mice, *Tp53* disruption rescues the exhaustion of *Rps19-*depleted HSCs (5, 57). A deficiency in the transcription factor MYSM1 in mice or humans impairs RP expression and causes TP53-dependent bone marrow failure (58, 59). Most importantly, germline activating *TP53* mutations cause a clinical phenotype of DBA (60, 61). The current work supports these previous studies and provides new experimental approaches to investigate mechanisms of TP53 activation resulting from RP deficiency and the resultant functional outcomes.

Several mechanisms could explain how *RPS19* haploinsufficiency causes TP53 activation during hematopoiesis. In cancer cell lines and animal models, RPS19 deficiency disrupts nucleolar ribosomal assembly, resulting in the accumulation of other RPs that diffuse into the nucleoplasm (24, 62). Free RPL5 and RPL11 can activate TP53 by sequestering its negative regulator, MDM2 (62). Other reported mechanisms for RP deficiency–induced TP53 activation include suppression of the kinases PIM1 and AKT, leading to MDM2 inhibition (63), increased *TP53* translation caused by free RPL26 and nucleolin (64), stabilization of TP53 by nucleoplasmic NOP53 (65), and a deficiency of GATA1, which may inhibit TP53 by physical association (19, 66, 67). A deficiency of RPS19 also impairs stem cell maintenance through TP53-independent mechanisms. For example, MDM2 promotes mesenchymal stem cell and cancer cell self-renewal by enhancing the activity of polycomb repressor complex 2, independent of TP53 (68). Therefore, in *RPS19*-mutated DBA, sequestration of MDM2 by free RPL5 or RPL11 could impair HSCs through multiple mechanisms.

Haploinsufficiency of *RPL5* or *RPL11*, the next most common DBA genes (1, 2), may impair hematopoiesis through different mechanisms than those associated with *RPS19* haploinsufficiency (23, 46, 69). Transcriptome analysis showed enrichment for IFN-α and -γ response pathways and depletion of GATA1 targets in erythroid progenitors from individuals with *RPS19*-mutated, but not *RPL5*- or *RPL11*-mutated, DBA (46). Moreover, erythroblasts from DBA patients with *RPL5* or *RPL11* mutations uniquely exhibit proteasomal degradation of HSP70 (69). Notably, *Rps19*-mutant mouse embryonic stem cells exhibit TP53 activation, while *Rpl5* mutants do not (46). Similarly, *Rpl11-*mutant mice exhibit DBA-like erythropoietic failure and impaired TP53 activation (70), consistent with knockdown experiments in different human cell lines (13, 44, 45, 71). However, TP53 activation was detected in erythroblasts generated from *RPL11*-depleted CD34^+^ cells (13) and in erythroid progenitors from patients with *RPL5*- or *RPL11*-mutated DBA (23). These apparent inconsistencies may represent species- and/or cell type–specific differences in responses to RP deficiencies and could potentially be resolved by creating and analyzing *RPL5*- or *RPL11*-disrupted CD34^+^ HSPCs using the approaches described here.

Our findings have clinical implications. The observation that *TP53* disruption restores bone marrow repopulation capacity to *RPS19^+/–^* HSCs raises the possibility that selective pressure could expand DBA patient HSPCs with somatic loss-of-function *TP53* mutations, analogous to what occurs in other bone marrow failure disorders, including Shwachman-Bodian-Diamond syndrome (72), Fanconi anemia (73), and dyskeratosis congenita (74). An increased rate of clonal hematopoiesis with *TP53* mutations has not been reported for DBA, although rates of myelodysplastic syndrome and myeloid leukemia are mildly elevated (75). Low-level premalignant *TP53* mutant clones have been shown to expand after allogeneic or autologous HSCT for sickle cell disease and myeloid leukemias (75, 76). In principle, this risk could be increased in older patients with DBA who undergo autologous gene therapy or allogeneic HSCT with reduced intensity conditioning. Therefore, it is important to evaluate patients with DBA for somatic *TP53* mutations and other clonal hematopoiesis-associated genes before and after therapeutic HSCT because the results of such studies may inform future therapies. Previous studies in mice (30, 77) and our current study with human cells show that normal or gene-corrected HSCs outcompete *RPS19^+/–^* HSCs after transplantation. These findings suggest that reduced-intensity bone marrow conditioning may be sufficient for full engraftment of allogeneic donor (78) or autologous gene-corrected HSCs, although this approach might be avoided if the DBA recipient harbors hematopoietic cell clones with somatic mutations of *TP53* or other leukemia-associated genes.

Our study has several limitations. First, the HSC defect in our model appears to be more severe that what occurs in DBA where bone marrow cellularity declines gradually in the second and third decades of life (5). This difference may be due to the enhanced ability of *RPS19^+/+^* HSPCs to outcompete *RPS19^+/–^* ones after xenotransplantation, impaired maintenance of human *RPS19^+/–^* HSPCs in a mouse bone marrow microenvironment, and/or stresses associated with HSC transplantation. Second, because *RPS19^+/–^* HSCs did not repopulate the bone marrow after xenotransplantation, we were unable to study the erythropoietic defect in vivo. Third, it is not clear whether the current model recapitulates the variable penetrance observed in *RPS19*-mutated DBA (1). Future studies using additional CD34^+^ HSPC donors are required to address this point.

In summary, our studies have shown that Cas9-mediated heterozygous *RPS19* mutations in healthy donor CD34^+^ HSPCs cause DBA-like erythroid defects and a TP53-dependent deficiency in bone marrow repopulation after xenotransplantation. Our findings define a robust and scalable experimental model for *RPS19*-mutated DBA, and provide potential insights into mechanisms of bone marrow failure that occurs in older affected patients. This experimental approach can be used for future therapeutic and mechanistic studies of DBA caused by mutations in *RPS19* and other RP genes.

## Methods

### Isolation and culture of CD34^+^ HSPCs.

Peripheral G-CSF–mobilized human mononuclear cells were collected from healthy adult volunteer donors (Key Biologics, Lifeblood). CD34^+^ HSPCs were enriched by immunomagnetic bead selection, using an AutoMACS instrument (Miltenyi Biotec) in accordance with the manufacturer’s instructions. The CD34^+^ cell percentage was at least 95%, as measured by flow cytometry. Isolated CD34^+^ HSPCs were cryopreserved and stored in liquid nitrogen until needed. Thawed cells were grown in culture at 37°C in 5% CO_2_ in HSPC maintenance medium (Supplemental Table 3), with the cell concentration being maintained between 0.2 × 10^6^ and 1.0 × 10^6^ cells/mL.

### In vitro hematopoietic differentiation of CD34^+^ HSPCs.

For erythroid differentiation, cells were grown in culture for 7 days at a density of 10^5^–10^6^ cells/mL in IMDM-based phase I medium and then for another 7 days in phase II erythroid medium. Myeloid differentiation was performed by seeding CD34^+^ HSPCs at a concentration of 1 × 10^4^ cells/mL in SFEM II medium supplemented with StemSpan Myeloid Expansion Supplement (Stem Cell Technologies, catalog 02693). Media and cytokines used for cell culture are described in Supplemental Table 3.

### Cytospin preparation.

Morphology of differentiated cells was assessed by depositing 1.5 × 10^5^ cells on glass slides by centrifugation at 250 rpm for 5 minutes, using a Cytospin 4 centrifuge (Thermo Fisher Scientific). Cells were then stained with May-Grünwald solution (Sigma-Aldrich, catalog MG1L-1L) for 2 minutes, rinsed in water, stained for 10 minutes with Giemsa solution (Sigma-Aldrich, catalog GS500), rinsed in water, and air dried, after which coverslips were mounted.

### CRISPR/Cas9 gene editing.

The sgRNAs used for genome editing are shown in Supplemental Table 1. RNP complex was prepared by incubating Cas9-3×NLS protein (from the St. Jude Protein Production Facility) and sgRNA (with 2′-O-methyl 3′ phosphorothioate modifications in the first and last 3 nucleotides [Synthego]) at a 1:3 molar ratio for 15 minutes at room temperature. Next, 2 × 10^5^ to 1 × 10^6^ CD34^+^ HSPCs were washed and resuspended in P3 buffer (Lonza, catalog V4LP-3002) before RNP was added for a final Cas9 concentration of 0.4 mg/mL and a total volume of 20 μL. For down-titration experiments, RNPs were diluted in P3 buffer to the specified final Cas9 concentration. For multiplex editing, RNPs targeting different genes were prepared separately and mixed together before being electroporated into cells. For transient TP53 inhibition, GSE56 mRNA (Cellscript) was added to the electroporation mix at a final concentration of 3 μg per 20 μL reaction (49). Electroporation was performed with a Lonza 4D-nucleofector (catalog AAF-1003X), using program DS-130, in accordance with the manufacturer’s instructions. After RNP treatment, cells were resuspended in HSPC maintenance medium.

### Measurement of on-target indel frequencies.

Three days after RNP electroporation, the targeted amplicons were generated using gene-specific primers with partial Illumina adapter overhangs (Supplemental Table 1) and sequenced on a MiSeq System (Illumina). Briefly, cell pellets were lysed and used to generate gene-specific amplicons with partial Illumina adapters in PCR 1. Amplicons were indexed in PCR 2 and pooled with other targeted amplicons for other loci to create sequence diversity. MyTaq DNA polymerase (Meridian Bioscience) or Platinum SuperFi DNA polymerase (Thermo Fisher Scientific) was used in PCR 1 for 35 cycles in accordance with the manufacturer’s recommendation. MyTaq polymerase was used in PCR 2, which consisted of 5 cycles. Additionally, 10% PhiX Sequencing Control V3 (Illumina) was added to the pooled amplicon library before running the sample on the MiSeq System to generate paired 2 × 250-bp reads. Samples were demultiplexed by using index sequences, FastQ files were generated, and NGS analysis was performed using CRIS.py (79). For analysis of human donor cells after transplantation into mice, background sequencing error rates were reduced by trimming 5′ and 3′ ends in CRIS.py. To determine the genotype of BFU-E colonies generated by gene-edited HSPCs, individual colonies were picked and the on-target indel frequency determined. Colonies with an *RPS19* indel frequency of less than 25% were graded as unedited wild-type cells, those with an indel frequency between 25% and 75% were graded as heterozygously edited cells, and those with an indel frequency greater than 75% were deemed to have undergone homozygous *RPS19* editing. An *RPS19* mutational profile was generated using CRISPResso2 with standard optional parameters (80).

### Xenotransplantation studies.

For xenotransplantation, 4 × 10^5^ to 5 × 10^5^ HSPCs were washed and resuspended in PBS with 2% fetal bovine serum (FBS) and then injected into the tail veins of 5- to 8-week-old female NSGW mice, which were bred in-house. Mice were euthanized and analyzed at 16 weeks after xenotransplantation. Recipient bone marrow cells were incubated with mouse- and human lineage–specific antibodies (Supplemental Table 4) and fractionated on a FACSAria II cell sorter (BD Biosciences). Indel frequencies were determined by NGS analysis of mouse bone marrow or purified human hematopoietic lineages.

### Western blot analysis.

Cell pellets were resuspended in PBS and lysed in 2× Laemmli sample buffer (Sigma-Aldrich, catalog S3401). Proteins were resolved by electrophoresis in a 4%–12% Bis-Tris Plus gel (Life Technologies, catalog NW04120) and transferred to a polyvinylidene difluoride (PVDF) membrane overnight. Membranes were blocked with 5% nonfat milk in TBST (Tris-buffered saline + 0.1% Tween 20). Membranes were incubated with primary antibody for 2 hours at room temperature, washed with TBST, and then incubated for 1 hour at room temperature with the secondary antibody, horseradish peroxidase–conjugated (HRP-conjugated) anti-rabbit IgG (Invitrogen, catalog 31460; 1:10,000 dilution). Immunoreactive material was visualized using Pierce ECL Plus Western Blotting Substrate (Thermo Fisher Scientific, catalog 32132) and imaged with a ChemiDoc Touch Imaging System (Bio-Rad). Quantitation was performed using Image Lab Software (Bio-Rad) by normalizing each RPS19 band to an actin loading control.

### Northern blot.

RNA was extracted from HSPCs using an RNeasy kit (Qiagen, catalog 74034) as per the manufacturer’s instructions. Following gel fractionation using a 1.5% formaldehyde-agarose gel, RNA was transferred to zeta-probe nylon membranes (Bio-Rad, catalog 1620153). ^32^P-labeled ITS1 probes were incubated with the membrane at 37°C overnight in hybridization buffer (Ambion, catalog AM8670) before phosphorimaging.

### Flow cytometry.

Erythroid cells were assessed by flow cytometry for the cell surface markers c-Kit, CD235a, CD71, CD41a, CD45RA, IL-3 receptor, CD105, and CD34 (Supplemental Table 4), and 7AAD (BD Biosciences, catalog 559925) was used for live/dead staining. Annexin V staining (BD Pharmingen, catalog 556547) was performed according to the manufacturer’s instructions. BrdU staining was performed by treating erythroid cells at different time points with 10 μM BrdU for 45 minutes and staining them with an FITC BrdU Flow Kit (BD Biosciences, catalog 559619) in accordance with manufacturer’s instructions. Myeloid cells were analyzed by flow cytometry on day 14 of culture, using antibodies against CD45, CD15, and CD33 (Supplemental Table 4). All flow cytometric analyses were performed with an LSRFortessa Cell Analyzer (BD Biosciences) and analyzed with FlowJo software (Tree Star).

### Methocult assay.

For each assay, 500–1000 CD34^+^ HSPCs were plated in 1 mL of methylcellulose media (Stem Cell Technologies, catalog 4435) in triplicate in 35-mm tissue culture dishes. Colonies were enumerated at 14 days after plating. Cells were pooled, washed with PBS, and counted using a NucleoCounter NC-3000 cytometer (ChemoMetec) to determine the cell count per colony.

### LV preparation and transduction.

LVs were prepared by the St. Jude Vector Core as described previously (81). CD34^+^ HSPCs were transduced, at a concentration of 2 × 10^6^ cells/mL, by treating with LVs at an MOI of 20, overnight for 16–20 hours. The transduction medium consisted of HSPC maintenance medium (Supplemental Table 3) supplemented with 1% human albumin (Grifols Biologicals), 10 μM prostaglandin E2 (Cayman Chemical), and 1 mg/mL LentiBOOST (Sirion Biotech) (82).

### Evaluation of VCN.

To determine the VCN, 1000 CD34^+^ cells were plated in 1 mL of methylcellulose medium (Stem Cell Technologies, catalog 4435) in triplicate and maintained in culture for 14 days. To determine the VCN from mouse bone marrow at 16 weeks after transplantation, 50,000 cells from mouse bone marrow were plated similarly in 1 mL of methylcellulose medium. After 14 days, colonies were pooled and genomic DNA was extracted using a DNeasy Blood and Tissue Kit (Qiagen). Genomic DNA was digested with *Msp*I restriction enzyme (New England Biolabs) and used as a template in ddPCR with primer-probe sets targeting the lentiviral Psi region and the human *RPP30* gene (Supplemental Table 1). Droplets were read using a QX200 Droplet Reader (Bio-Rad), and data were analyzed using QuantaSoft software v.1.7.4.0917 (Bio-Rad). VCNs per diploid genome were calculated as the ratio of Psi copies to every 2 copies of *RPP30*.

### RT-ddPCR.

RNA was extracted from RNP-treated cells by using a RNeasy Mini Kit (Qiagen), and the concentration of RNA was determined using a NanoDrop spectrophotometer (Thermo Fisher Scientific). The change in *CDKN1A* expression was determined by using the One-Step RT-ddPCR Advanced Kit for Probes (Bio-Rad, catalog 1864021) in accordance with the manufacturer’s protocol, with the *CDKN1A* primers/probe (Bio-Rad, assay 10031252, assay ID: dHSACPE5052298) and with the *RPP30* primers/probe (Bio-Rad, assay 10031255, assay ID: dHSAcpe5038241) as controls. Briefly, droplets were prepared using an Automated Droplet Generator (Bio-Rad), and PCR amplification was performed as follows: a first step at 50°C for 60 minutes, a second step at 95°C for 10 minutes, a third step at 95°C for 30 seconds followed by 55°C for 1 minute (for 40 cycles), with a final step at 98°C for 10 minutes. Droplets were analyzed as described above.

### Statistics.

All experiments were replicated at least twice with CD34^+^ HSPCs from different donors. Data from all experiments are presented as the mean ± standard deviation (SD) unless otherwise noted in the figure legend. Statistical analyses were performed using GraphPad Prism v9.2.0 or the open-source software R (www.R-project.org). Normality was determined with the Shapiro-Wilk test, and subsequently analyzed with a 2-tailed Student’s *t* test or Wilcoxon’s rank-sum test. Longitudinal data were analyzed using linear mixed-effects model analysis. For multiple comparison, *P* values were adjusted by the Holm-Bonferroni method. *P* values less than 0.05 were considered to indicate statistical significance.

### Study approval.

NSGW (NOD, SCID, *Il2rg^−/−^*, *Kit^W41/W41^*) mice (83) were housed and handled in strict accordance with the NIH *Guide for the Care and Use of Laboratory Animals* (National Academies Press, 2011). Animal experiments were carried out in accordance with a protocol (Genetic Tools for the Study of Hematopoiesis) approved by the institutional animal care and use committee of St. Jude Children’s Research Hospital.

## Author contributions

SVB and MJW designed the experiments, analyzed the data, and wrote the manuscript. SVB performed gene editing, in vitro differentiation studies, and xenotransplantation studies with help from TM, YY, KDM, and MALO. YJ performed RT-ddPCR. SRE performed Northern blotting. JSSL assisted with statistical analysis. JSY, LB, and MWW provided conceptual advice and technical expertise. MJW supervised the study. All authors discussed the results and contributed to preparing the manuscript.

## Supplementary Material

Supplemental data

## Version 1 11/22/2022

In-Press Preview

## Version 2 01/10/2023

Electronic publication

## References

[1] Da Costa L (2020). Diamond-Blackfan anemia. Blood.

[2] Da Costa L (2018). An update on the pathogenesis and diagnosis of Diamond-Blackfan anemia. F1000Res.

[3] Simkins A (2017). Diamond-Blackfan anemia predisposing to myelodysplastic syndrome in early adulthood. JCO Precis Oncol.

[4] Vlachos A (2012). Incidence of neoplasia in Diamond Blackfan anemia: a report from the Diamond Blackfan Anemia Registry. Blood.

[5] Giri N (2000). Clinical and laboratory evidence for a trilineage haematopoietic defect in patients with refractory Diamond-Blackfan anaemia. Br J Haematol.

[6] Iskander D (2019). Impaired cellular and humoral immunity is a feature of Diamond-Blackfan anaemia; experience of 107 unselected cases in the United Kingdom. Br J Haematol.

[7] Sankaran VG (2012). Exome sequencing identifies GATA1 mutations resulting in Diamond-Blackfan anemia. J Clin Invest.

[8] Kim AR (2017). Functional selectivity in cytokine signaling revealed through a pathogenic EPO mutation. Cell.

[9] Ulirsch JC (2018). The genetic landscape of Diamond-Blackfan anemia. Am J Hum Genet.

[10] Khajuria RK (2018). Ribosome levels selectively regulate translation and lineage commitment in human hematopoiesis. Cell.

[11] Perdahl EB (1994). Erythroid failure in Diamond-Blackfan anemia is characterized by apoptosis. Blood.

[12] Iskander D (2015). Elucidation of the EP defect in Diamond-Blackfan anemia by characterization and prospective isolation of human EPs. Blood.

[13] Moniz H (2012). Primary hematopoietic cells from DBA patients with mutations in RPL11 and RPS19 genes exhibit distinct erythroid phenotype in vitro. Cell Death Dis.

[14] Ohene-Abuakwa Y (2005). Two-phase culture in Diamond Blackfan anemia: localization of erythroid defect. Blood.

[15] O’Brien KA (2017). Molecular convergence in ex vivo models of Diamond-Blackfan anemia. Blood.

[16] Boussaid I (2021). Integrated analyses of translatome and proteome identify the rules of translation selectivity in RPS14-deficient cells. Haematologica.

[17] Horos R (2012). Ribosomal deficiencies in Diamond-Blackfan anemia impair translation of transcripts essential for differentiation of murine and human erythroblasts. Blood.

[18] Taylor AM (2020). Calmodulin inhibitors improve erythropoiesis in Diamond-Blackfan anemia. Sci Transl Med.

[19] Ludwig LS (2014). Altered translation of GATA1 in Diamond-Blackfan anemia. Nat Med.

[20] Yang Z (2016). Delayed globin synthesis leads to excess heme and the macrocytic anemia of Diamond Blackfan anemia and del(5q) myelodysplastic syndrome. Sci Transl Med.

[21] Doty RT (2022). Studies of a mosaic patient with DBA and chimeric mice reveal erythroid cell-extrinsic contributions to erythropoiesis. Blood.

[22] Dutt S (2011). Haploinsufficiency for ribosomal protein genes causes selective activation of p53 in human erythroid progenitor cells. Blood.

[23] Iskander D (2021). Single-cell profiling of human bone marrow progenitors reveals mechanisms of failing erythropoiesis in Diamond-Blackfan anemia. Sci Transl Med.

[24] Bursać S (2012). Mutual protection of ribosomal proteins L5 and L11 from degradation is essential for p53 activation upon ribosomal biogenesis stress. Proc Natl Acad Sci U S A.

[25] Jaako P (2015). Disruption of the 5S RNP-Mdm2 interaction significantly improves the erythroid defect in a mouse model for Diamond-Blackfan anemia. Leukemia.

[26] Wilkes MC (2020). Diamond Blackfan anemia is mediated by hyperactive Nemo-like kinase. Nat Commun.

[27] Doulatov S (2017). Drug discovery for Diamond-Blackfan anemia using reprogrammed hematopoietic progenitors. Sci Transl Med.

[28] Alter BP (2010). Malignancies and survival patterns in the National Cancer Institute inherited bone marrow failure syndromes cohort study. Br J Haematol.

[29] Jaako P (2011). Mice with ribosomal protein S19 deficiency develop bone marrow failure and symptoms like patients with Diamond-Blackfan anemia. Blood.

[30] Flygare J (2008). Gene therapy of Diamond Blackfan anemia CD34(+) cells leads to improved erythroid development and engraftment following transplantation. Exp Hematol.

[31] Zivny J (2003). Diamond blackfan anemia stem cells fail to repopulate erythropoiesis in NOD/SCID mice. Blood Cells Mol Dis.

[32] McGowan KA, Mason PJ (2011). Animal models of Diamond Blackfan anemia. Semin Hematol.

[33] Miyake K (2005). Development of cellular models for ribosomal protein S19 (RPS19)-deficient Diamond-Blackfan anemia using inducible expression of siRNA against RPS19. Mol Ther.

[34] Ebert BL (2005). An RNA interference model of RPS19 deficiency in Diamond-Blackfan anemia recapitulates defective hematopoiesis and rescue by dexamethasone: identification of dexamethasone-responsive genes by microarray. Blood.

[35] Garcon L (2013). Ribosomal and hematopoietic defects in induced pluripotent stem cells derived from Diamond Blackfan anemia patients. Blood.

[36] Yan H (2021). Comprehensive phenotyping of erythropoiesis in human bone marrow: evaluation of normal and ineffective erythropoiesis. Am J Hematol.

[37] Kim JH (2011). High cleavage efficiency of a 2A peptide derived from porcine teschovirus-1 in human cell lines, zebrafish and mice. PLoS One.

[38] Milone MC, O’Doherty U (2018). Clinical use of lentiviral vectors. Leukemia.

[39] Flygare J (2007). Human RPS19, the gene mutated in Diamond-Blackfan anemia, encodes a ribosomal protein required for the maturation of 40S ribosomal subunits. Blood.

[40] Bae S (2014). Cas-OFFinder: a fast and versatile algorithm that searches for potential off-target sites of Cas9 RNA-guided endonucleases. Bioinformatics.

[41] Matsson H (2004). Targeted disruption of the ribosomal protein S19 gene is lethal prior to implantation. Mol Cell Biol.

[42] Hamaguchi I (2003). Proliferation deficiency of multipotent hematopoietic progenitors in ribosomal protein S19 (RPS19)-deficient diamond-Blackfan anemia improves following RPS19 gene transfer. Mol Ther.

[43] Hamaguchi I (2002). Gene transfer improves erythroid development in ribosomal protein S19-deficient Diamond-Blackfan anemia. Blood.

[44] Teng T (2013). Loss of tumor suppressor RPL5/RPL11 does not induce cell cycle arrest but impedes proliferation due to reduced ribosome content and translation capacity. Mol Cell Biol.

[45] Fumagalli S (2012). Suprainduction of p53 by disruption of 40S and 60S ribosome biogenesis leads to the activation of a novel G2/M checkpoint. Genes Dev.

[46] Singh SA (2014). p53-Independent cell cycle and erythroid differentiation defects in murine embryonic stem cells haploinsufficient for Diamond Blackfan anemia-proteins: RPS19 versus RPL5. PLoS One.

[47] Conti A, Di Micco R (2018). p53 activation: a checkpoint for precision genome editing?. Genome Med.

[48] Tothova Z (2017). Multiplex CRISPR/Cas9-based genome editing in human hematopoietic stem cells models clonal hematopoiesis and myeloid neoplasia. Cell Stem Cell.

[49] Schiroli G (2019). Precise gene editing preserves hematopoietic stem cell function following transient p53-mediated DNA damage response. Cell Stem Cell.

[50] Ferrari S (2020). Efficient gene editing of human long-term hematopoietic stem cells validated by clonal tracking. Nat Biotechnol.

[51] Le Goff S (2021). p53 activation during ribosome biogenesis regulates normal erythroid differentiation. Blood.

[52] Giri N (2000). Clinical and laboratory evidence for a trilineage haematopoietic defect in patients with refractory Diamond-Blackfan anaemia. Br J Haematol.

[53] Armstrong RN (2020). Erythropoiesis failure in Diamond-Blackfan anemia starts at the oligopotent common myeloid and megakaryocyte-erythroid progenitor stage. Blood.

[54] Santucci MA (1999). Long-term bone marrow cultures in Diamond-Blackfan anemia reveal a defect of both granulomacrophage and erythroid progenitors. Exp Hematol.

[55] Jain AK, Barton MC (2018). p53: emerging roles in stem cells, development and beyond. Development.

[56] Cho IJ (2019). Mechanisms, hallmarks, and implications of stem cell quiescence. Stem Cell Reports.

[57] Casadevall N (1994). Age-related alterations in erythroid and granulopoietic progenitors in Diamond-Blackfan anaemia. Br J Haematol.

[58] Belle JI (2020). MYSM1 maintains ribosomal protein gene expression in hematopoietic stem cells to prevent hematopoietic dysfunction. JCI Insight.

[59] Belle JI (2015). p53 mediates loss of hematopoietic stem cell function and lymphopenia in Mysm1 deficiency. Blood.

[60] Toki T (2018). De novo mutations activating germline TP53 in an inherited bone-marrow-failure syndrome. Am J Hum Genet.

[61] Fedorova D (2022). De novo TP53 germline activating mutations in two patients with the phenotype mimicking Diamond-Blackfan anemia. Pediatr Blood Cancer.

[62] Zhang Y (2003). Ribosomal protein L11 negatively regulates oncoprotein MDM2 and mediates a p53-dependent ribosomal-stress checkpoint pathway. Mol Cell Biol.

[63] Sagar V (2016). PIM1 destabilization activates a p53-dependent response to ribosomal stress in cancer cells. Oncotarget.

[64] Chen J (2012). Interactions of nucleolin and ribosomal protein L26 (RPL26) in translational control of human p53 mRNA. J Biol Chem.

[65] Lee S (2012). Nucleolar protein GLTSCR2 stabilizes p53 in response to ribosomal stresses. Cell Death Differ.

[66] Trainor CD (2009). GATA-1 associates with and inhibits p53. Blood.

[67] Rio S (2019). Regulation of globin-heme balance in Diamond-Blackfan anemia by HSP70/GATA1. Blood.

[68] Wienken M (2016). MDM2 associates with polycomb repressor complex 2 and enhances stemness-promoting chromatin modifications independent of p53. Mol Cell.

[69] Gastou M (2017). The severe phenotype of Diamond-Blackfan anemia is modulated by heat shock protein 70. Blood Adv.

[70] Morgado-Palacin L (2015). Partial loss of Rpl11 in adult mice recapitulates Diamond-Blackfan anemia and promotes lymphomagenesis. Cell Rep.

[71] Sloan KE (2013). The 5S RNP couples p53 homeostasis to ribosome biogenesis and nucleolar stress. Cell Rep.

[72] Kennedy AL (2021). Distinct genetic pathways define pre-malignant versus compensatory clonal hematopoiesis in Shwachman-Diamond syndrome. Nat Commun.

[73] Abo-Elwafa HA (2011). The prognostic value of p53 mutation in pediatric marrow hypoplasia. Diagn Pathol.

[74] Schaefer EJ, Lindsley RC (2018). Significance of clonal mutations in bone marrow failure and inherited myelodysplastic syndrome/acute myeloid leukemia predisposition syndromes. Hematol Oncol Clin North Am.

[75] Ghannam JY (2020). Baseline TP53 mutations in adults with SCD developing myeloid malignancy following hematopoietic cell transplantation. Blood.

[76] Wong TN (2015). Role of TP53 mutations in the origin and evolution of therapy-related acute myeloid leukaemia. Nature.

[77] Dahl M (2021). Bone marrow transplantation without myeloablative conditioning in a mouse model for Diamond-Blackfan anemia corrects the disease phenotype. Exp Hematol.

[78] Koyamaishi S (2021). Reduced-intensity conditioning is effective for hematopoietic stem cell transplantation in young pediatric patients with Diamond-Blackfan anemia. Bone Marrow Transplant.

[79] Connelly JP, Pruett-Miller SM (2019). CRIS.py: a versatile and high-throughput analysis program for CRISPR-based genome editing. Sci Rep.

[80] Clement K (2019). CRISPResso2 provides accurate and rapid genome editing sequence analysis. Nat Biotechnol.

[81] Bauler M (2020). Production of lentiviral vectors using suspension cells grown in serum-free media. Mol Ther Methods Clin Dev.

[82] Jang Y (2020). Optimizing lentiviral vector transduction of hematopoietic stem cells for gene therapy. Gene Ther.

[83] Rahmig S (2016). Improved human erythropoiesis and platelet formation in humanized NSGW41 mice. Stem Cell Reports.

