# Supplementary material for: An RPS19-edited model for Diamond-Blackfan anemia reveals TP53-dependent impairment of hematopoietic stem cell activity
Source: JCI Insight. 2023 Jan 10;8(1):e161810. doi: 10.1172/jci.insight.161810 (PMC9870085; doi:10.1172/jci.insight.161810)
Supplement: Supplemental data [file jciinsight-8-161810-s102.pdf]

Supplemental Table 1. Nucleic acid sequences of single-guide RNAs, primers, and probes

Assay	Name	Sequences (5′ to 3′)
**sgRNAs**	AAVS1	ACCCCACAGTGGGGCCACTA
	RPS19.1	TACCCCCAGCTTCCACAGCG
	RPS19.2	TGTAAAAGACGTGAACCAGC
	RPS19.3	CTGACGTCCCCCATAGATCT
	TP53	TCCTCAGCATCTTATCCGAG
**NGS primers**	AAVS1 forward	AGTCTTCTTCCTCCAACCCGGGCCC
	AAVS1 reverse	CCTGCCAAGCTCTCCCTCCCAGGAT
	RPS19.1 and 3 forward	GTGGGTGAGGAGAGGGGGCTGTCAG
	RPS19.1 and 3 reverse	TCAATGCAGCCCCCTCTACCCTGCT
	RPS19.2 forward	TGAAGGGGCCGTGGAAGTAACG
	RPS19.2 reverse	GCCTCGCCTGCCAGGGACCG
	TP53 forward	GGCGCTGCCCCCACCATGAG
	TP53 reverse	CTGGAGGGCCACTGACAACCACCCT
**ddPCR primers and probes**	Psi forward	ACTTGAAAGCGAAAGGGAAAC
	Psi reverse	CACCCATCTCTCTCCTTCTAGCC
	Psi probe	5′FAM-AGCTCTCTCGACGCAGGACTCGGC
	RPP30 forward	GCGGCTGTCTCCACAAGT
	RPP30 reverse	GATTTGGACCTGCGAGCG
	RPP probe	5′HEX-CTGACCTGAAGGCTCT
**Northern blot probe**	ITS1 probe	CCTCGCCCTCCGGGCTCCGTTAATGA

Supplemental Table 2: Reference sequence and top 10 most frequent edited reads for three different sgRNAs targeting RPS19. sgRNA-binding sequences are in red, insertions are in blue, and deletions are represented as dashes.

**Name**	**Sequence**	**Read frequency (%)**	**Read count**	**Frameshift**
Reference sequence (RPS19.1)	CACTACCCCCAGCTTCCACAGCGCGGCACCTGTACCTCCG			
Edited reads	CACTACCCCCAGCTTCCACAAGCGCGGCACCTGTACCTCC	35.2	345	Yes
	CACTACCCCCAGCTTCC - - - - - - - - - - - - - TGTACCTCCG	3.6	35	Yes
	CACTACCCCCAGCTTCCAC - - - - - - - - - - CTGTACCTCCG	3.1	30	Yes
	CACTACCCCCAGC - - - - - - - - - - - GGCACCTGTACCTCCG	1	10	Yes
	CACTACCCCCAGCT - - - - - - - - - - - - - - - - - - - GTACCTCCG	0.9	9	Yes
	CACTACC - TCCG	0.6	6	Yes
	CACTACCCCCAGCTTCCAC - - - - - - - - - - - - - - - - CTCCG	0.5	5	Yes
	CACTACCCCCAGCTTCCACAAAGCGCGGCACCTGTACCTC	0.5	5	Yes
	CACTACCCCCAGCTTCCACAAGCGCGGCGCCTGTACCTCC	0.4	4	Yes
	CACTACCCCCAGCTTCCA - -GCGCGGCACCTGTACCTCCG	0.4	4	Yes
Reference sequence (RPS19.2)	TACTGTAAAAGACGTGAACCAGCAGGAGTTCGTCAGAGCT			
Edited reads	TACTGTAAAAGACGTGAAC - AGCAGGAGTTCGTCAGAGCT	23.02	325	Yes
	TACTGTAAAAGACGTGAACCCAGCAGGAGTTCGTCAGAGC	8.6	122	Yes
	TACTGTAAAAGACGTGAACC - GCAGGAGTTCGTCAGAGCT	7.1	100	Yes
	TACTGTAAAAGACGTGAA - - - GCAGGAGTTCGTCAGAGCT	2.6	36	No
	TACTGTAAAAGACGTGAACCAAGCAGGAGTTCGTCAGAGC	2.3	33	Yes
	TACTGTAAAAGACGTGAACCCCAGCAGGAGTTCGTCAGAG	2.2	31	Yes
	TACTGTAAAAGACGTGAA - - AGCAGGAGTTCGTCAGAGCT	2.1	29	Yes
	TACTGTAAAAGAC - - - - - - - AGCAGGAGTTCGTCAGAGCT	2	28	Yes
	TACTGTAAAAGACGTGA - - - - - - - - - - GTTCGTCAGAGCT	1.9	27	Yes
	TACTGTAAAAGACGTGA - - CAGCAGGAGTTCGTCAGAGCT	1.8	26	Yes
Reference sequence (RPS19.3)	GGTTGGCTCCATGACCAAGATCTATGGGGGACGTCAGAGA			
Edited reads	GGTTGGCTCCATGACCAAGAATCTATGGGGGACGTCAGAG	12.3	44	Yes
	GGTTGGCTCCATGACCAAGA - - - - - GGGGGACGTCAGAGA	9.8	35	Yes
	GGTTGGCTCCATGACCAAGA - - - - TGGGGGACGTCAGAGA	8.4	30	Yes
	GGTTGGCTCCATGACCAAGA - - TATGGGGGACGTCAGAGA	7.5	27	Yes
	GGTTGGCTCCATGACCAAGATTCTATGGGGGACGTCAGAG	6.7	24	Yes
	GGTTGGCTCCATGACCAAGACTCTATGGGGGACGTCAGAG	4.2	15	Yes
	GGTTGGCTCCATGACCAAGA - - - ATGGGGGACGTCAGAGA	3.3	12	No
	GGTTGGCTCCATGACCAAGA - CTATGGGGGACGTCAGAGA	2.2	8	Yes
	GGTTGGCTCCATGACCAAGAGTCTATGGGGGACGTCAGAG	1.7	6	Yes
	GGTTGGCTCCATGACCAAGA - - - - - - GGGGACGTCAGAGA	1.4	5	No

Supplemental Table 3. Media and cytokines

Medium	Component	Manufacturer	Catalog #	Final Concentration
**HSPC maintenance**	X-Vivo 10 (base)	Lonza	BEBP02-055Q	
	Human stem cell factor	R&D Systems	255-SC/CF	100 ng/mL
	Thrombopoietin	R&D Systems	288-TP/CF	100 ng/mL
	FLT-3 ligand	R&D Systems	3088-FK/CF	100 ng/mL
**Erythroid differentiation**	Common to all phases			
	IMDM (base)	Thermo Fisher	12440061	
	Human male AB plasma	SeraCare	1810-0001	2%
	Human AB serum	Atlanta Biologicals	S40110	3%
	Heparin	Sagent Pharmaceuticals	NDC 25021-401-02	3 IU/mL
	EPO	Amgen	NDC 55513-144-01	3 IU/mL
	Penicillin–Streptomycin	Thermo Fisher	15070063	Penicillin 50 U/mL Streptomycin 50 μg/mL
**Erythroid differentiation (Phase I)**	Add the following to the common components:			
	Human holo-transferrin	Millipore	T0665	200 μg/mL
	Human stem cell factor	R&D Systems	255-SC/CF	10 ng/mL
	Human IL-3	R&D Systems	203-IL/CF	1 ng/mL
**Erythroid differentiation (Phase II)**	Add the following to the common components:			
	Human holo-transferrin	Millipore	T0665	200 μg/mL
	Human stem cell factor	R&D Systems	255-SC/CF	10 ng/mL

Supplemental Table 4. Antibodies used in flow cytometry panels and Western blots

Panel	Antibody	Clone	Manufacturer	Catalog #
**Mouse bone marrow studies**	BV786 Anti-Mouse CD45	30-F11	BD Biosciences	564225
	BV605 Anti-Human CD45	HI30	BD Biosciences	564047
	PE-Cy7 Anti-Human CD33	P67.6	BD Biosciences	333946
	PE Anti-Human CD19	4G7	BD Biosciences	349209
	Alexa Fluor 700 Anti-Human CD34	581	BD Biosciences	561440
	PerCP-Cy5.5 Anti-Mouse Ter119	TER-119	BD Biosciences	560512
	APC Anti-Human CD235	GA-R2 (HIR2)	BD Biosciences	551336
	APC-Cy7 Anti-Human CD3	SK7	BD Biosciences	557832
**Erythroid differentiation**	BV421 Anti-CD235a	GA-R2 (HIR2)	BD Biosciences	562938
	BV510 Anti-CD41a	HIP8	BD Biosciences	563250
	PE Anti-CD117	A3C6E2	BioLegend	323408
	PE-CF594 Anti-CD105	266	BD Biosciences	562380
	PE-Cy7 Anti-IL3R	6H6	BioLegend	306010
	APC Anti-CD34	Clone 582	BD Biosciences	555824
	APC-H7 Anti-CD71	M-A712	BD Biosciences	563671
**Myeloid differentiation**				
	BV605 Anti-CD45	HI30	BD Biosciences	564047
	Alexa Fluor 488 Anti-CD15	W6D3	BioLegend	323010
	PE-Cy7 Anti-CD33	P67.6	BioLegend	366618
**Western Blot**				
	RPS19	EPR10423	Abcam	181365
	TP53	DO-1	BD Biosciences	554293
	CDKN1A	12D1	CST	2947
	GFP	N/A	Abcam	AB6556

Supplemental figures:

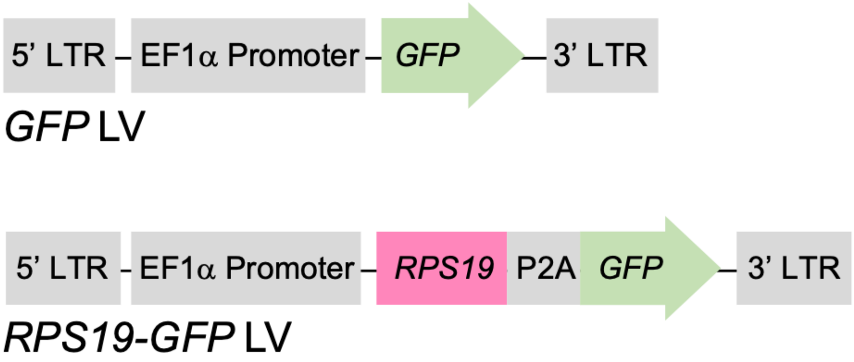

Supplemental Figure 1 (related to main Figure 1). Design of lentiviral vectors used in the study. (A) Diagram of third-generation self-inactivating LVs expressing *GFP* alone or *RPS19* + *GFP* separated by auto-cleaving P2A sequence, driven by EF1α core promoter.

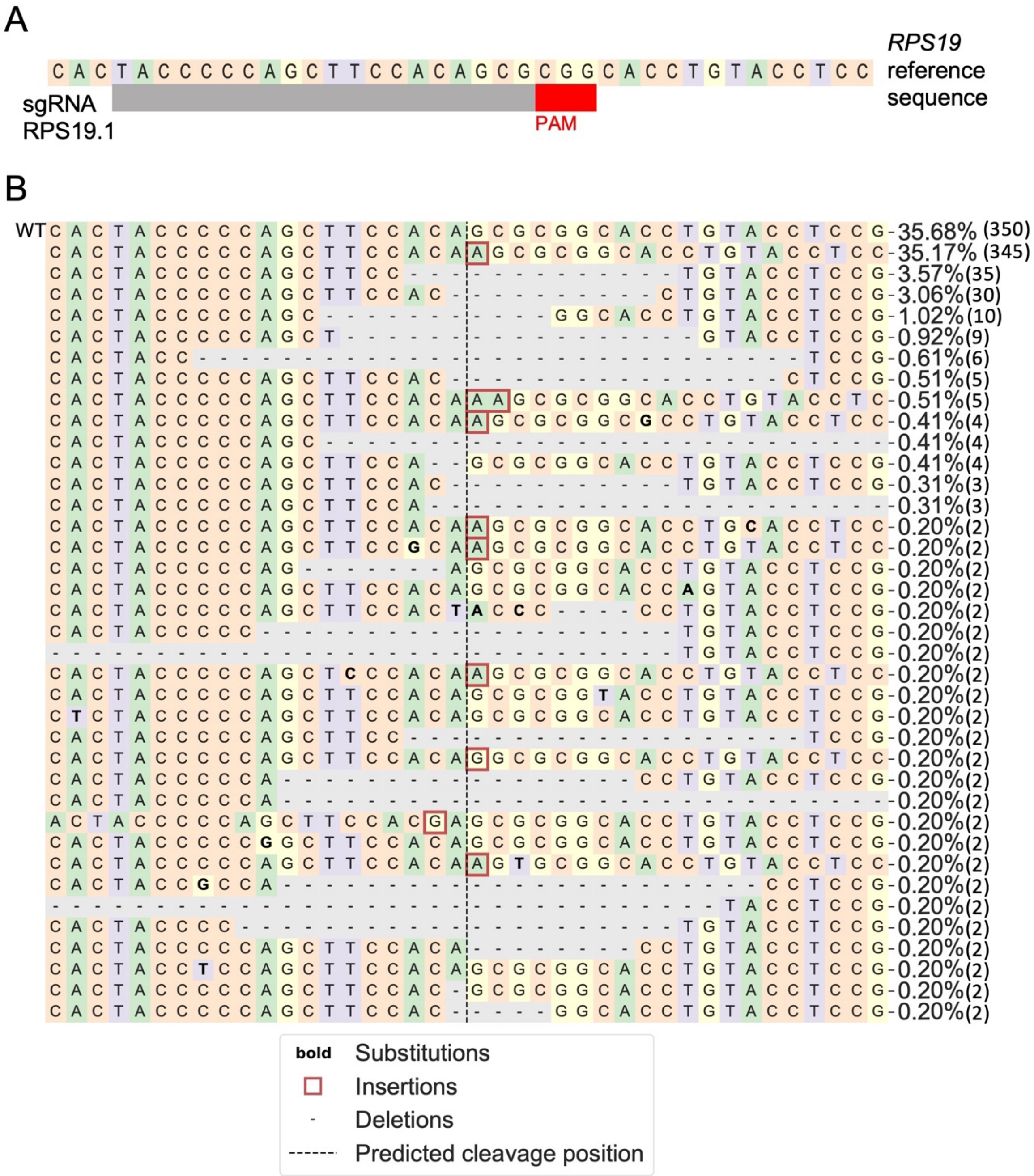

Supplemental Figure 2 (related to main Figure 1). *RPS19* indels in CD34+ hematopoietic stem and progenitor cells (HSPCs) 3 days after editing with Cas9 + RPS19.1 single-guide RNA (sgRNA) ribonucleoprotein (RNP). (A) *RPS19* reference sequence with complementary sgRNA RPS19.1 represented below**. (B)** Results of indel analysis performed on day 0 according to the scheme shown in main Figure 1B. The unedited wild-type (WT) sequence is shown at top. The percentage of each indel is shown at right, with the NGS read counts in parentheses.

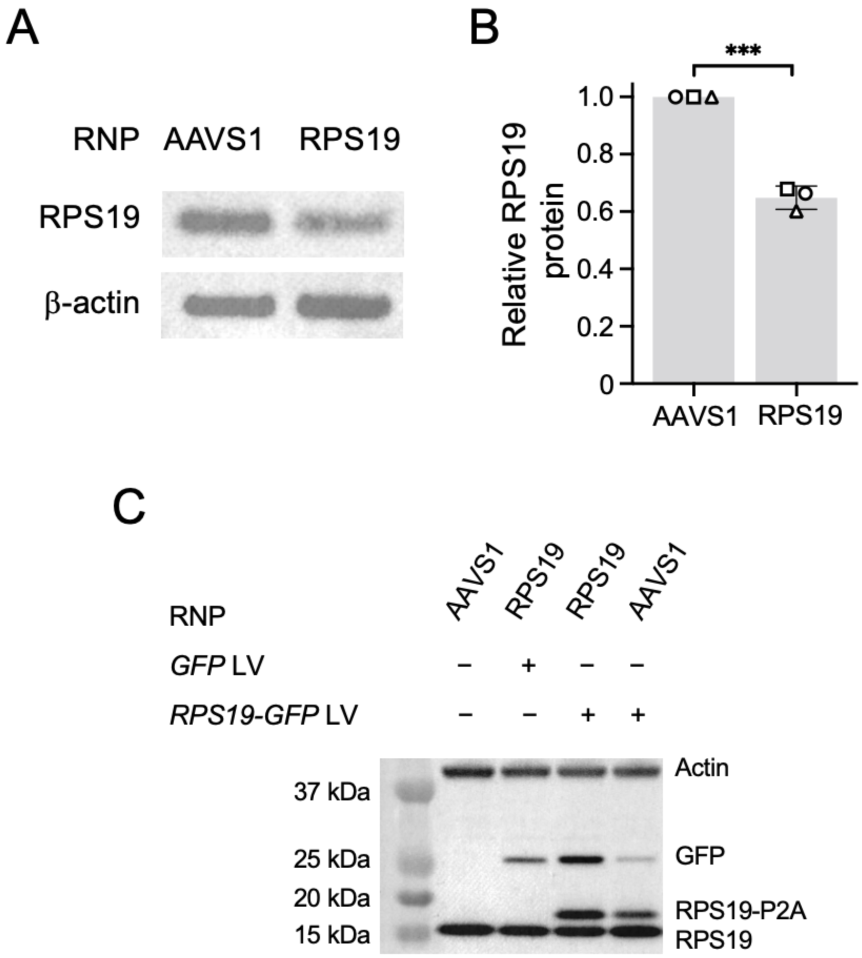

Supplemental Figure 3 (related to main Figure 1). Reduced RPS19 protein and rescue by LV-derived RPS19 in *RPS19*-disrupted CD34⁺ HSPCs. Cells were edited with RPS19.1 RNP, transduced with or without LV, and analyzed 3 days after editing. **(A)** Representative Western blot showing RPS19 protein expression. **(B)** Relative RPS19 protein levels (normalized to actin) after editing with AAVS1 or RPS19.1 RNP. The bar chart shows the mean ± SD of 3 independent experiments (unpaired, 2-tailed Student *t*-test). **(C)** Western blot showing RPS19, GFP and actin loading control.

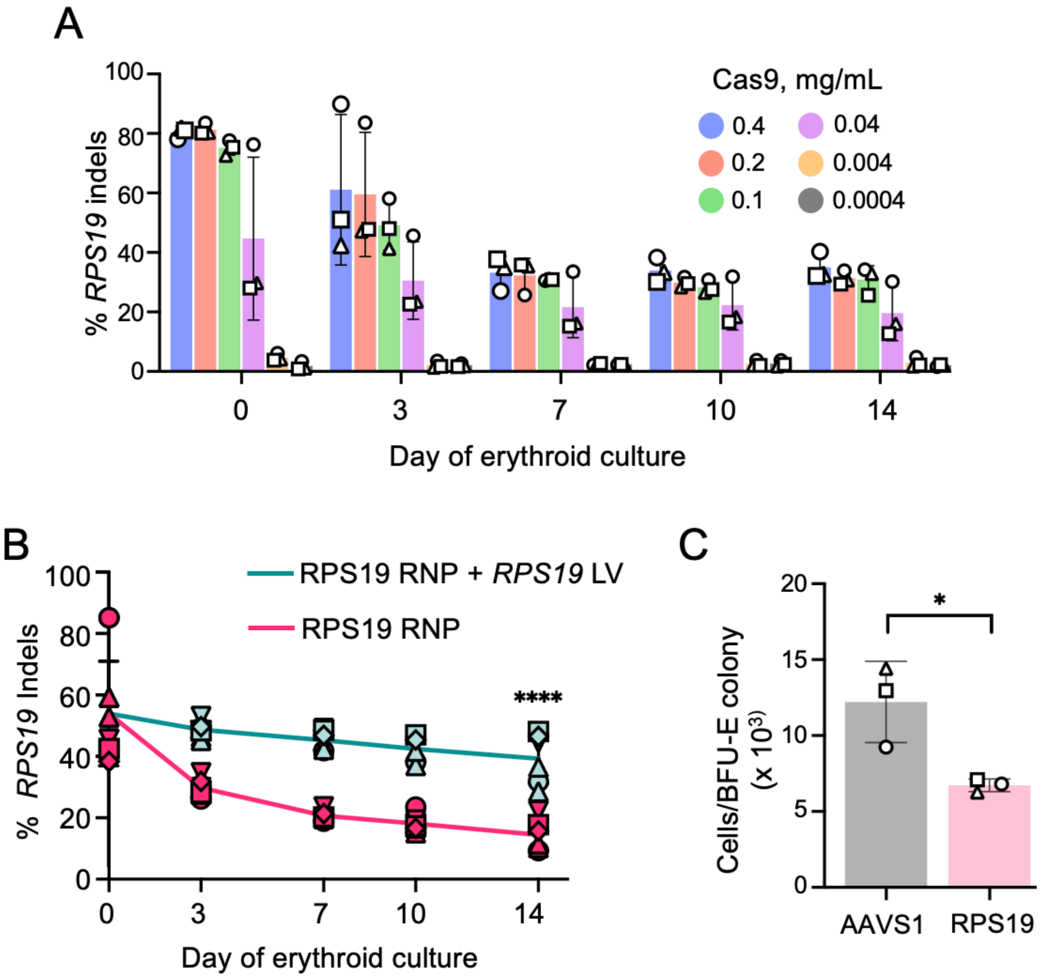

Supplemental Figure 4 (related to main Figure 2). Transduction with *RPS19* lentiviral vector (LV) partially rescues the erythropoietic defect of *RPS19*$^{+/-}$ HSPCs. (A) CD34^{+} HSPCs were edited with the indicated concentrations of RPS19 RNP on day −3. On day 0, cells were switched to erythroid differentiation medium and indel frequencies were determined serially. Bar chart shows mean ± SD, with each symbol representing different HSPC donors. **(B)** RPS19 RNP–treated cells, ± *RPS19* LV transduction, were generated as shown in main Figure 1B then grown in culture in erythroid medium. The graph shows the *RPS19* indel frequency versus time. The data points are the mean ± SD of 6 biological replicates performed using 3 different CD34^{+} HSPC donors (2 experiments per donor), represented by different symbols (Mixed model-effects analysis). **(C)** Cells per BFU-E colony generated by *AAVS1* or *RPS19*-targeted CD34^{+} HSPCs. Each symbol represents data from a different CD34^{+} HSPC donor, with the bar chart showing the mean ± SD (unpaired, 2-tailed Student *t*-test).

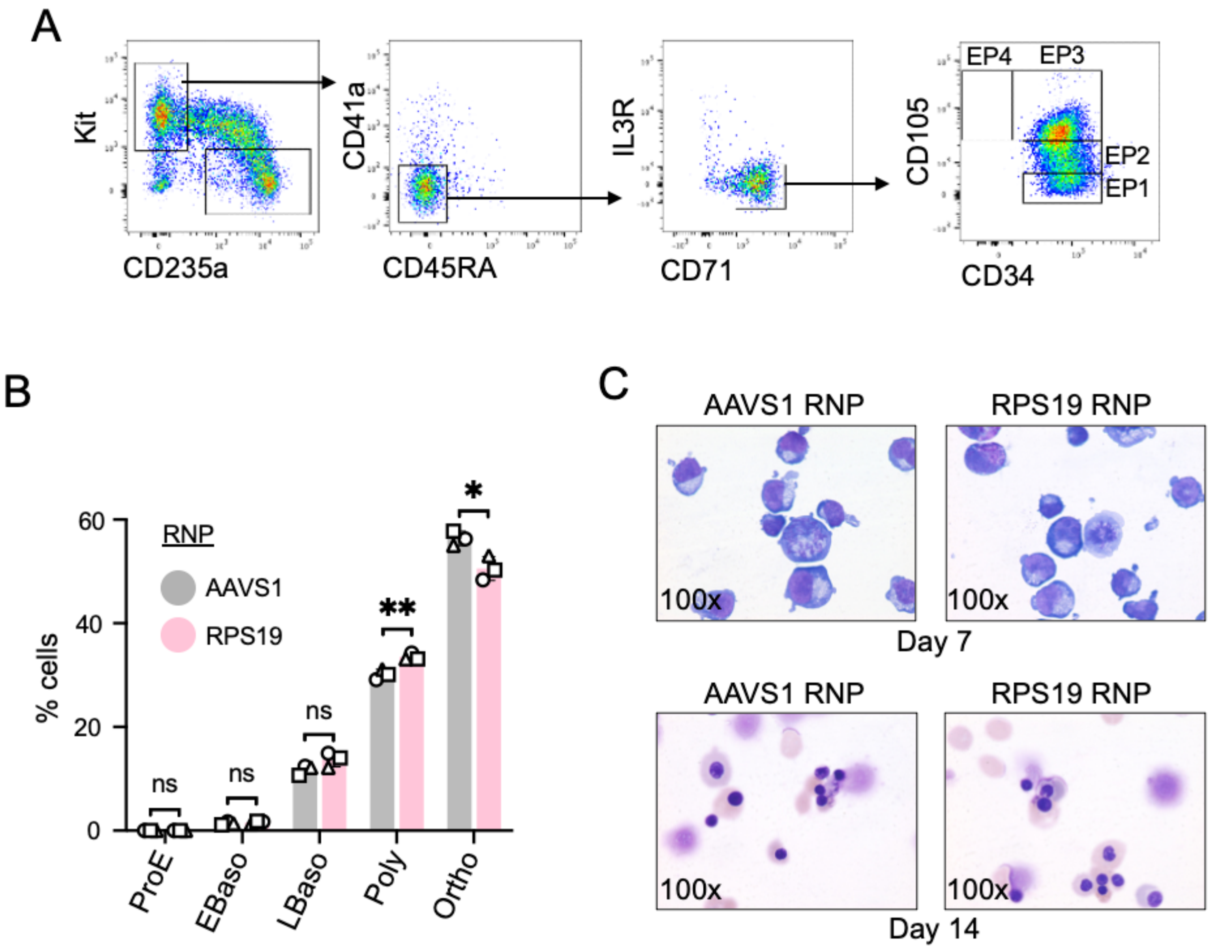

Supplemental Figure 5 (related to main Figure 3). *RPS19*$^{+/-}$ HSPCs exhibit erythroid maturation defect. (A) Representative flow cytometry plots of normal CD34^{+} HSPC in vitro erythroid differentiation showing the gating strategy for BFU-E (erythroid progenitor [EP] 1) and CFU-E (EP 2–4). **(B)** Effects of *RPS19* disruption on terminal erythroid maturation of CD34^{+} HSPCs. Cells were analyzed at day 14 of erythroid culture and gated as shown in Figure 3B. The bar chart shows the mean ± SD. Each symbol represents data from a different CD34^{+} HSPC donor (unpaired, 2-tailed Student *t*-test). **(C)** May–Grunwald and Giemsa–stained erythroblasts at days 7 and 14 of erythroid differentiation. Images were obtained with a Nikon Eclipse NI microscope, using a Nikon DS Qi2 camera.

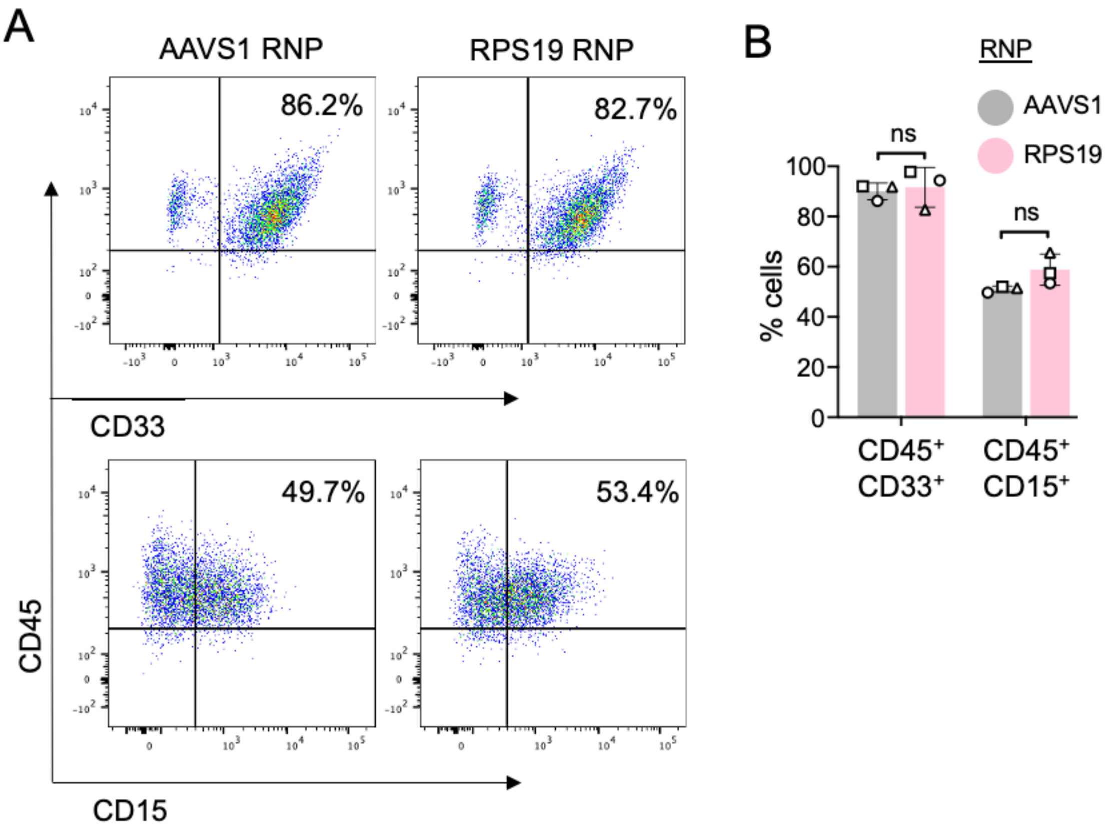

Supplemental Figure 6 (related to main Figure 3). *RPS19* disruption does not impair in vitro myeloid differentiation. $CD34^+$ HSPCs were treated with AAVS1 or RPS19 RNP as described in Figure 1B, grown in myeloid differentiation medium for 14 days, then analyzed for maturation markers. **(A)** Representative flow cytometry plots after staining with antibodies against myeloid surface markers. **(B)** Summary of multiple experiments performed as described above, using $CD34^+$ HSPCs from 3 different donors. The bar chart shows the mean ± SD (unpaired, 2-tailed Student *t*-test).

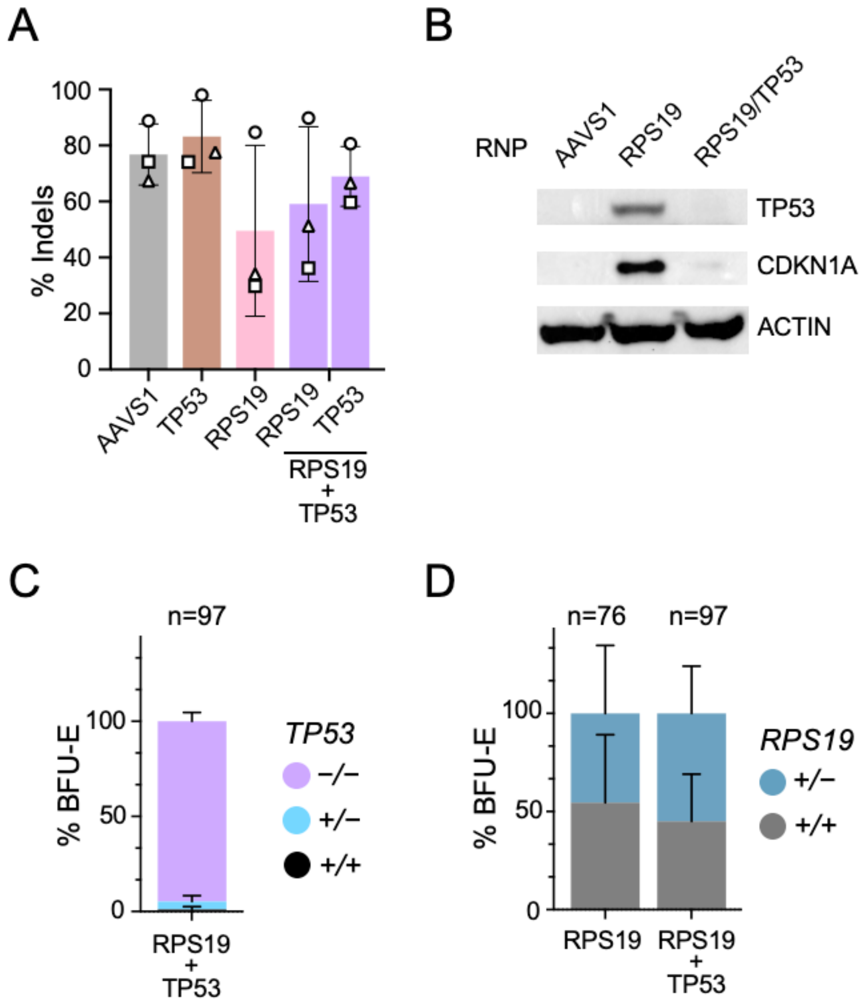

Supplemental Figure 7 (related to main Figure 4). Multiplex editing of *RPS19* and *TP53* in CD34⁺ HSPCs. CD34⁺ HSPCs were edited with AAVS1 or RPS19.1 ± TP53 RNPs according to the protocol in Figure 1B. **(A)** Indel frequency corresponding to each targeting RNP at 3 days after electroporation. Each symbol represents data from different CD34⁺ cell donors. **(B)** Western blot showing TP53, CDKN1A and actin loading control. **(C, D)** Genotype distributions in BFU-E colonies generated from CD34⁺ HSPCs treated with RNPs targeting *RPS19* ± *TP53*. n = total colonies analyzed from biological replicate experiments using 2 different CD34⁺ HSPC donors. All bar charts show the mean ± SD.

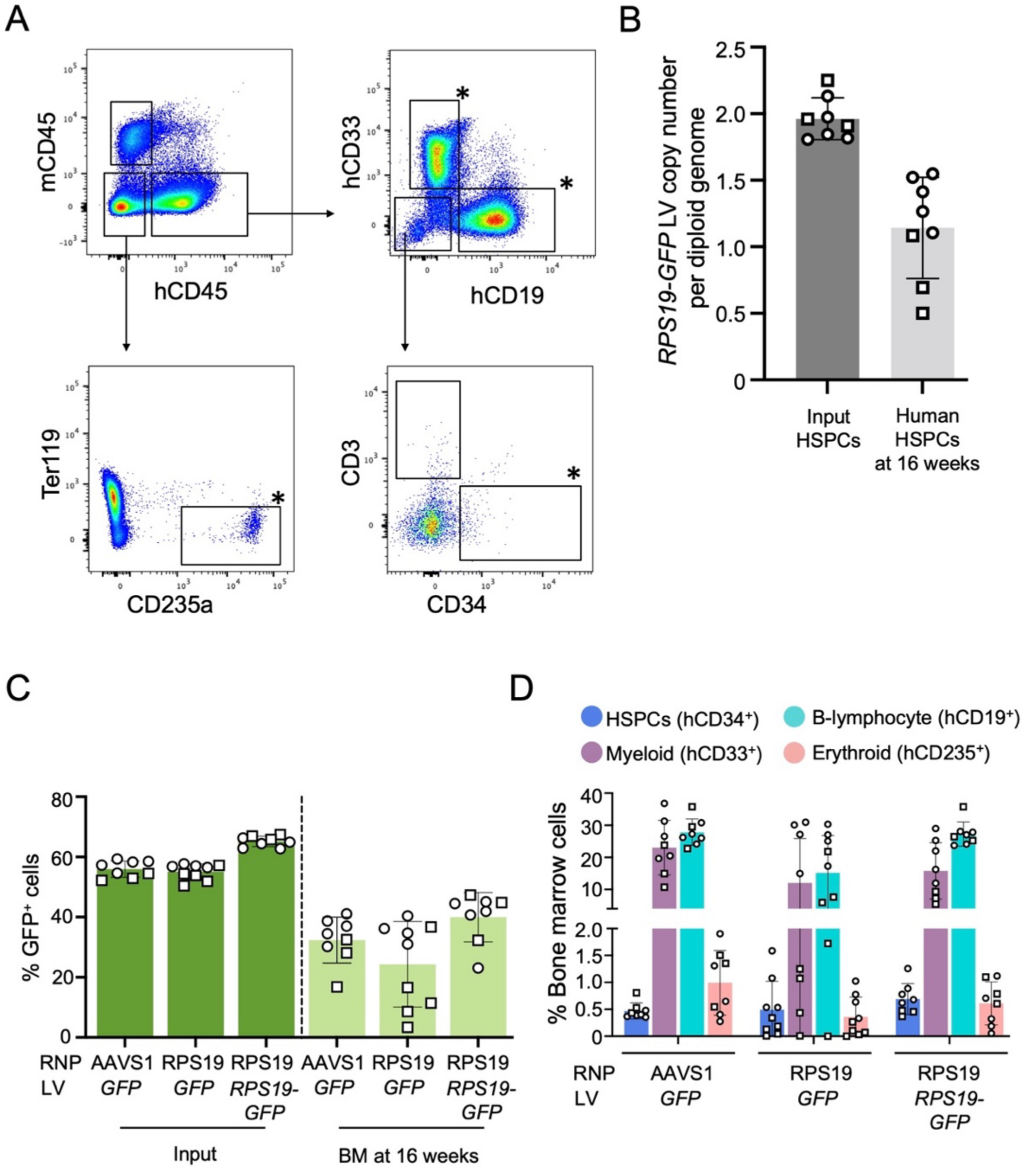

Supplemental Figure 8 (related to main Figure 5). *RPS19* expressing LV partially rescues bone marrow repopulation defect of $RPS19^{+/-}$ HSPCs. Normal donor HSPCs were treated with RPS19.1 RNP ± RPS19-GFP LV then transplanted into NSGW mice, which underwent necropsy 16 weeks later, according to the protocol in Figure 4A. **(A)** Representative flow cytometry plots showing the gating strategy used to assess human HSPC repopulation. Asterisks indicate populations that were analyzed for *RPS19* indels shown in main Figures 4D and 5C. **(B)** *RPS19-GFP* LV copy number per diploid genome in input HSPCs and after xenotransplantation. **(C)** LV-transduced (%GFP^+) cells in input $CD34^+$ HSPCs and 16 weeks after xenotransplantation. **(D)** Percentages of human $CD34^+$ HSPCs and their progeny in recipient mouse bone marrow at 16 weeks. All bar charts show the mean ± SD, with each symbol representing data from a different $CD34^+$ cell donor.

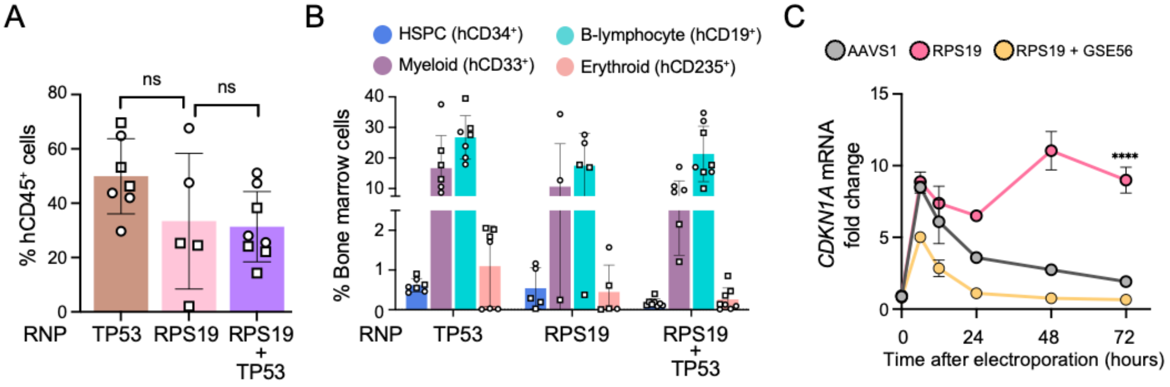

Supplemental Figure 9 (related to main Figure 6). Engraftment of *RPS19/TP53* multiplex-edited human cells after xenotransplantation. Normal human HSPCs were edited and transplanted into NSGW mice, as described in main Figure 4A. Necropsy was performed at 16 weeks, and recipient bone marrow populations were analyzed by flow cytometry. **(A)** Percentage of human CD45$^+$ cells in recipient bone marrow at 16 weeks post transplant. Data were analyzed by ANOVA test and pairwise testing was performed with Tuckey's adjustment for multiple comparison. **(B)** Percentage of human CD34$^+$ HSPCs and their differentiated progeny in recipient bone marrow. The corresponding *RPS19* indel frequencies in each population are shown in main Figure 6C. **(C)** *CDKN1A* mRNA fold change over time, relative to the level in unedited cells. Each data point represents the mean ± SD of 3 biological replicate experiments using CD34$^+$ cells from different donors. Linear mixed-effects model approach was used to test for statistical significance. Bar charts show the mean ± SD of the data, with each symbol representing data from a different CD34$^+$ cell donor.
